# Orosomucoid 1 Ameliorates Temporomandibular Joint Osteoarthritis by Maintaining Cartilage Homeostasis

**DOI:** 10.1002/advs.202500028

**Published:** 2025-06-29

**Authors:** Dahe Zhang, Yuxin Zhang, Simo Xia, Lu Chen, Pei Shen, Chi Yang

**Affiliations:** ^1^ Department of Oral Surgery Shanghai Ninth People's Hospital Shanghai Jiao Tong University School of Medicine College of Stomatology Shanghai Jiao Tong University National Center for Stomatology National Clinical Research Center for Oral Diseases No. 639, Zhi zao ju Road Shanghai 200011 China

**Keywords:** cartilage, orosomucoid, osteoarthritis, temporomandibular joint, vimentin

## Abstract

Temporomandibular joint osteoarthritis (TMJOA) is one of the most complex temporomandibular disorders. Cartilage matrix degradation results in the infiltration of nerves, blood vessels, and inflammatory cells, which disrupts chondrocyte function. Therapeutic strategies for TMJOA designed to maintain cartilage homeostasis remain largely unknown. Here, it is reported that orosomucoid 1 (ORM1) attenuated TMJOA progression by maintaining cartilage homeostasis. It is demonstrated that ORM1 is down‐regulated in the synovial fluid of patients with TMJOA and condylar cartilage of unilateral anterior crossbite (UAC) rats. Administration of ORM1 protein significantly inhibited cartilage matrix degradation and alleviated TMJOA progression in UAC rats. At the mechanistic level, ORM1 binds to vimentin (VIM), a type III intermediate filament cytoskeletal protein, and inhibits the mitogen‐activated protein kinase (MAPK) pathway, thereby significantly decreasing cartilage matrix degradation mediated through inhibiting the cartilage degradation markers matrix metalloproteinase 13 (MMP13) and MMP3, and maintaining cartilage homeostasis. Notably, inhibition of VIM in vivo also markedly improved TMJOA progression, including cartilage degradation and subchondral bone destruction. In conclusion, these findings demonstrate the important functions of ORM1 in maintaining cartilage homeostasis via suppressing VIM/MAPK/MMP signaling and suggest that ORM1 is a promising target for therapeutic intervention in TMJOA.

## Graphical abstract

The present study identified the key protein ORM1 through the proteomic analysis of synovial fluid samples from patients with temporomandibular joint osteoarthritis (TMJOA), and demonstrated the important role of ORM1 in maintaining cartilage homeostasis via the suppression of VIM/MAPK/MMP signalling. It suggests that ORM1 is a promising target for therapeutic intervention in TMJOA.

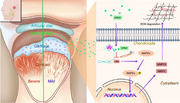

## 1 Introduction

Temporomandibular joint osteoarthritis (TMJOA) is a complicated temporomandibular disorder with a high incidence and can cause several problems, including pain and dysfunction.^[^ ^1^ ^]^ However, the mechanism of TMJOA remains unclear.^[^ ^2^ ^]^ The characteristics of TMJOA include synovitis, cartilage degeneration, and subchondral bone remodeling.^[^ ^3^ ^]^ Considering the uniqueness of condylar cartilage in mandibular development, cartilage degeneration in TMJOA has been widely investigated,^[^ ^4^ ^]^ and maintaining condylar cartilage homeostasis is considered the key strategy in treating TMJOA.^[^ ^4^, ^5^ ^]^

Disruption of cartilage homeostasis in TMJOA can be attributed to several factors, such as chondrocyte apoptosis,^[^ ^6^ ^]^ abnormal autophagy,^[^ ^7^ ^]^ and most importantly, degenerative changes in the extracellular matrix (ECM).^[^ ^8^ ^]^ Matrix metalloproteinases (MMPs) play an important role in ECM degradation, and their expression accelerates the progression of TMJOA.^[^ ^9^ ^]^ Thus, targeting MMPs is considered a potential therapeutic strategy for TMJOA.^[^ ^10^ ^]^

Apart from internal changes, surrounding structures and components, especially synovial fluid (SF), can directly influence the disease.^[^ ^4^, ^11^ ^]^ In our previous study, a proteomic analysis of SF samples from patients with anterior disc displacement (ADD)‐related TMJOA was performed to gain insight into the pathogenesis of cartilage degeneration and subchondral bone disruption in TMJOA at the protein level.^[^ ^11^ ^]^ In the present study, the analysis of 63 SF samples from patients with TMJOA showed that orosomucoid 1 (ORM1) ameliorates TMJOA by maintaining cartilage homeostasis.

ORM1, also known as α1‐1‐acid glycoprotein 1, is a glycoprotein, predominantly synthesized in the liver and detectable in blood plasma.^[^ ^12^ ^]^ Because ORM1 is acute‐phase protein, its levels in the blood increase in certain diseases, including inflammation, infection, and cancer.^[^ ^13^ ^]^ A key role of ORM1 is as a carrier protein in the blood, transporting various endogenous and exogenous substances, especially drugs.^[^ ^13b^ ^]^ Notably, ORM1 plays an immunomodulatory role by interacting with the immune system components and modulating inflammatory responses, and several studies have reported that ORM1 has an anti‐inflammatory effect in inflammation.^[^ ^14^ ^]^ Remarkably, it has been reported that serum ORM levels in patients with active knee OA are lower than those in patients with nonactive OA.^[^ ^15^ ^]^ However, to our knowledge, the role of ORM1 in the regulation of cartilage homeostasis in TMJOA has not been investigated.

In the present study, we aimed to explore the role of ORM1 in condylar cartilage in the development of TMJOA and to identify the underlying mechanisms using SF samples from patients with TMJOA, TMJOA rat models, and chondrocyte models. Our results revealed that ORM1 ameliorates TMJOA by maintaining cartilage homeostasis, which is achieved through binding to vimentin (VIM), thereby inhibiting VIM expression and the downstream mitogen‐activated protein kinase (MAPK) pathway, including extracellular signal‐regulated kinase (ERK), c‐Jun N‐terminal kinase (JNK) and p38 mitogen‐activated protein kinase (p38), and eventually significantly decreasing MMP3 and MMP13 mediated ECM degradation. Our study provides a novel perspective for therapeutic intervention in TMJOA by maintaining cartilage homeostasis and indicates that ORM1 is a promising therapeutic target.

## 2 Experimental Section

### 2.1 Ethical Statement

The primary human tissues were approved by the Human Research Ethics Committee of Shanghai Ninth People's Hospital, Shanghai Jiao Tong University School of Medicine (approval no. SH9H‐2020‐T7‐1) and conformed to the principles of the Declaration of Helsinki. Written informed consent was obtained from all participants and their guardians. Experiments on rats complied with the National Institutes of Health Guide for the Care and Use of Laboratory Animals, and were approved by the Institutional Animal Care and Use Committee of Shanghai Ninth People's Hospital (approval no. SH9H‐2023‐A197‐SB).

### 2.2 Patients, Clinical Information, and Samples

Patients diagnosed with ADD‐related TMJOA between January 2018 and January 2020 at the Shanghai Ninth People's Hospital were recruited. Information regarding age, gender, bruxism, pain, maximum interincisal opening (MIO), and TMJ magnetic resonance imaging (MRI) was collected. The detailed clinical data were shown in Table S1 (Supporting Information). TMJ MRI was performed for all patients using a 3.0T MR scanner (Ingenia; Philips Healthcare Systems) with a 6‐channel dS Flex M surface coil receiver.^[^ ^16^ ^]^ Proton‐density‐weighted imaging sequences were obtained in the oblique sagittal plane, with the patient's mouth closed, and T2‐weighted images were acquired with the patient's mouth maximally open. According to the TMJ‐ADD treatment protocol,^[^ ^17^ ^]^ patients with failed conservative treatments were recommended to undergo arthroscopic discopexy. SF samples were obtained from patients who agreed to undergo the surgery and provided informed consent. The sampling process was as follows: After administering a local anesthetic, 1 mL normal saline (NS) was injected into the upper TMJ space using a 21‐gauge needle for 30s elapse, and this procedure was repeated five times to obtain the SF samples.

Patients were classified into distinct groups according to OA severity determined on the basis of TMJ MRI results, following Yang's classification.^[^ ^18^ ^]^ In detail, mild or localized resorption without loss of height was described as mild OA; moderate resorption with reduced height was described as moderate OA; and a small condyle, significant resorption with loss of cortical bone integrity, or complete resorption was described as severe OA.

**Figure 1:**
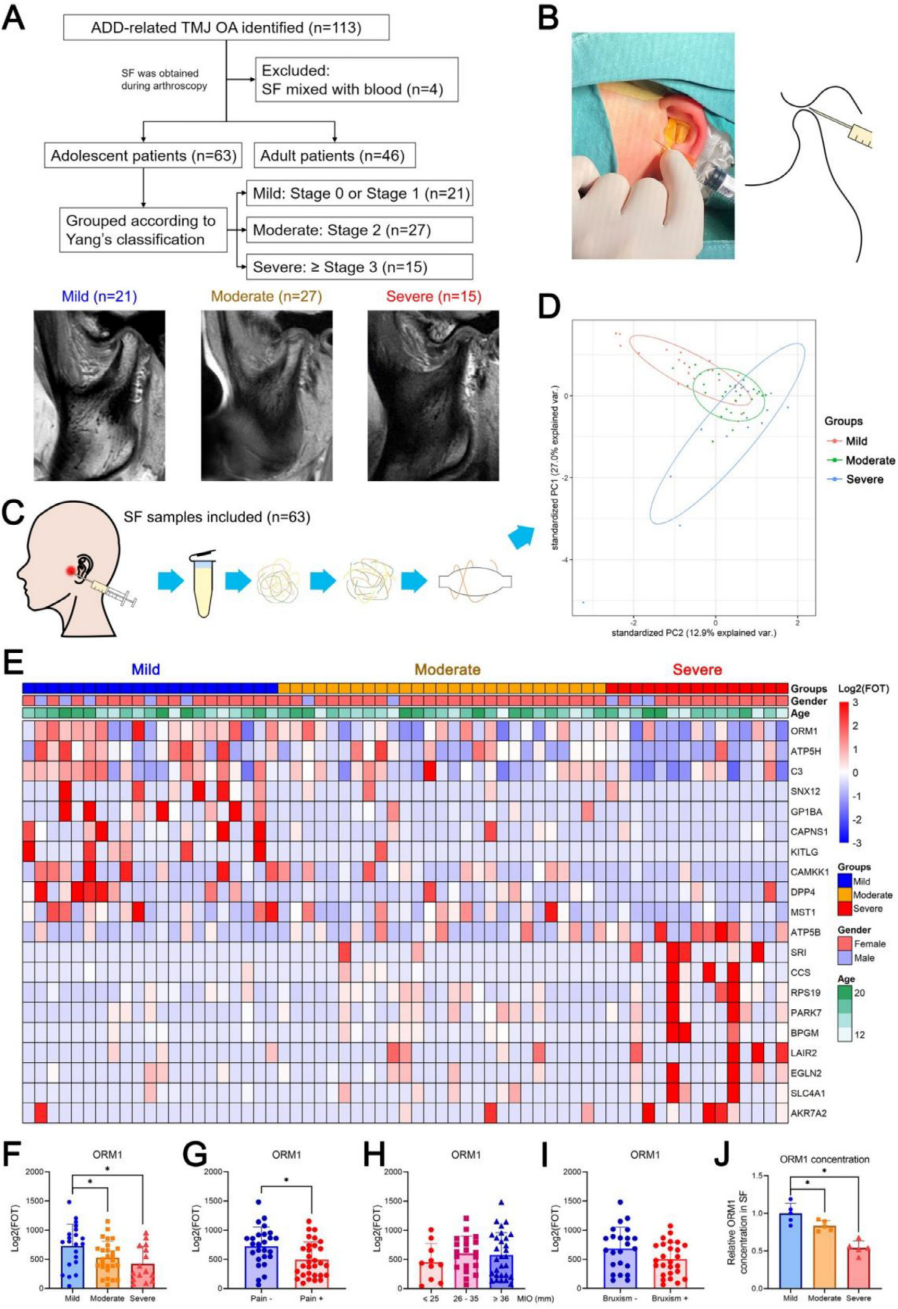
Proteomic analysis of the synovial fluid (SF) samples from patients with different stages of temporomandibular joint osteoarthritis (TMJOA). A) Flow diagram for patient inclusion and grouping. B) Photograph showing joint fluid collection during arthroscopic surgery. C) Flow diagram for the proteomic analysis of SF samples. D) Principal component analysis of SF samples. E) Top 10 genes that showed an upward trend and top 10 genes that showed a downward trend in the three groups. F) Orosomucoid 1 (ORM1) expression in patients with different grades of TMJOA, including Mild (n = 21), Moderate (n = 27), and Severe (n = 15) groups. Data is expressed as mean ± SD and analyzed using one‐way analysis of variance (ANOVA) followed by Tukey's post‐hoc test. G) ORM1 expression in patients with pain (n = 30) or without pain (n = 26). Data is expressed as mean ± SD and analyzed using unpaired Student's t test (two‐tailed). H) ORM1 expression in patients with different degrees of maximum interincisal opening (MIO). Data in MIO ≤ 25 mm (n = 10), MIO 26–35 mm (n = 20), and MIO ≥ 36 mm (n = 29) groups are expressed as mean ± SD and analyzed using one‐way ANOVA followed by Tukey's post‐hoc test. I) ORM1 expression in patients with sleep bruxism (n = 26) or without sleep bruxism (n = 23). Data is expressed as mean ± SD and analyzed using unpaired Student's t test (two‐tailed). J) ORM1 concentration in SF samples detected by ELISA assay in Mild (n = 5), Moderate (n = 5), and Severe (n = 5) groups. Data is expressed as mean ± SD and analyzed using one‐way ANOVA followed by Tukey's post‐hoc test. FOT, fraction of the total; *p < 0.05.

### 2.3 Proteomic Analysis

Proteomic analysis was performed using previously reported methods.^[^ ^11^ ^]^ SF samples were lysed and loaded with protein supernatants into 10 kDa filtration devices (Microcon; Millipore), filtered, and digested with trypsin. Peptides were extracted and dried. The samples were analyzed by liquid chromatography mass spectrometry (LC‐MS/MS) using an EASY‐nLC 1200 ultrahigh‐pressure system (Thermo) coupled via a nanoelectrospray ion source (Thermo) to a Fusion Lumos Orbitrap (Thermo). Data‐independent acquisition mass spectrometry was performed, and the data were analyzed using DIA‐NN (version 1.7.0). A label‐free, intensity‐based absolute quantification approach was used to perform the label‐free protein quantifications. The fraction of the total (FOT) represented the normalized abundance of a protein across samples.

### 2.4 Pathway Enrichment Analysis and Weighted Gene Co‐Expression Network Analysis

Differentially expressed proteins and differentially expressed genes (DEGs) were calculated by the *limma* R package. Gene Oncology (GO) term and Kyoto Encyclopedia of Genes and Genomes (KEGG) pathway enrichment analyses were performed using the *org.Hs.eg.db*, *clusterProfiler*, and *tidyverse* packages in R version 4.1.0 (www.r‐project.org),^[^ ^19^ ^]^ or Metascape (https://metascape.org/). Weighted gene co‐expression network analysis (WGCNA)^[^ ^20^ ^]^ was used to identify differentially co‐expressed gene modules. The association between a module and clinical information is signified by the eigengenes of each module. The genes in the modules were further analyzed using pathway enrichment analysis.

### 2.5 Enzyme‐Linked Immunosorbent Assay

Enzyme‐linked immunosorbent assay (ELISA) was performed to measure the concentration of ORM1 in SF samples by an ELISA kit (KE00137; Proteintech). The standard curve was created using the supplier's human ORM1 protein. Then, the SF samples were diluted, and the assay was performed according to the manufacturer's protocol. To avoid the influence of SF volume, the ratio of ORM1 concentration to each SF volume was calculated, and the relative ORM1 concentration ratio of the SF samples was analyzed.

### 2.6 Cell Lines and Culture Conditions

The human chondrocyte cell line C28/I2, chondrosarcoma cell line SW1353, and embryonic kidney cell line HEK‐293T were used in the experiments. C28/I2 cells and SW1353 cells were cultivated in Ham's F‐12K (Kaighn's) medium (F‐12K; Gibco) supplemented with 10% fetal bovine serum (FBS; Gibco). HEK‐293T cells were cultivated in Dulbecco's Modified Eagle Medium (Gibco) supplemented with 10% FBS. The cells were cultivated at 37 °C in 5% CO_2_.

### 2.7 Cell Proliferation Assay

Cell proliferation activity was assessed using a Cell Counting Kit‐8 assay (CCK8; Dojindo). In detail, C28/I2 cells were seeded in 96‐well plates (10 000 cells in 100 µL F‐12K with 10% FBS per well). The cells were treated with graded concentrations (50–400 µg mL^−1^) of ORM1 protein (Sigma–Aldrich). Then, on three consecutive days (days 1, 2, and 3) at the same time, 10 µL CCK8 reagent was added to each well. After incubation for 2 h, a microplate reader (Molecular Devices) was used to detect the absorbance at 450 nm.

### 2.8 Subcellular Localization of Exogenous ORM1

The fluorescein isothiocyanate (FITC) labelled ORM1 or BSA was made by Youke Biological Technology (Shanghai). The C28/I2 cells were incubated with FITC labelled BSA (control) or FITC‐labelled ORM1 without or with 100‐fold excess of unlabelled ORM1 for 1 h. Then, the cells were fixed with 4% paraformaldehyde, and stained with 4',6‐diamidino‐2‐phenylindole (DAPI). Images were taken using a confocal laser scanning microscope (Leica).

### 2.9 Temporomandibular Joint Osteoarthritis Model and Treatment

Rats were acquired from the Central Laboratory of Shanghai Ninth People's Hospital. Two batches of female Sprague−Dawley (SD) rats aged 4 weeks were randomly divided into different treatment groups. In these groups, unilateral anterior crossbite (UAC) occlusion was induced using a previously reported method to generate the TMJOA model rats^[^ ^21^ ^]^; At week 0, the rats were anesthetized using 1% pentobarbital (0.35 mL/100 g) and subjected to UAC construction. The rats were weighed every week. Subsequently, the rats were subjected to the treatments described below. After a treatment period of 2 weeks or 5 weeks, rats were euthanized at week 3 or week 6, respectively, and the condyles were harvested.

In the first batch, forty rats were divided into eight groups: I. control groups treated with 50 µL normal saline (NS) for 2 weeks (*n* = 5) and 5 weeks (*n* = 5), II. UAC groups treated with 50 µL NS for 2 weeks (*n* = 5) and 5 weeks (*n* = 5), III. UAC + 100 µg mL^−1^ ORM1 groups treated with 50 µL 100 µg/mL ORM1 for 2 weeks (*n* = 5) and 5 weeks (*n* = 5), and IV. UAC + 200 µg mL^−1^ ORM1 groups treated with 50 µL 200 µg mL^−1^ ORM1 for 2 weeks (*n* = 5) and 5 weeks (*n* = 5).

In the second batch, 20 µL 1 × 10^10^ vg mL^−1^ type 2 adeno‐associated viruses (AAV2) obtained from HANBIO (China) were locally injected into each rat TMJ capsule. Thirty rats were divided into six groups: I. AAV2‐siControl groups treated with AAV2‐siControl at week ‐2, and euthanized at week 3 (*n* = 5) and week 6 (*n* = 5), II. UAC + AAV2‐siControl groups treated with AAV2‐siControl at week ‐2, and then subjected to UAC construction at week 0 for 3 weeks (*n* = 5) and 6 weeks (*n* = 5), and III. UAC + AAV2‐siVIM groups were treated with AAV2‐siVIM at week ‐2, and then subjected to UAC construction at week 0 for 3 weeks (*n* = 5) and 6 weeks (*n* = 5).

### 2.10 Micro‐Computed Tomography

One of the two condyles from each rat was fixed in 4% polyformalin and underwent assessed using a microcomputed tomography (micro‐CT) system (SkyScan‐1176, Bruker), with a resolution of 8.96 µm, X ray energy of 50 kV, 450 µA, and 0.4° rotation step (180° angular range). The acquired images of condyles were reconstructed and viewed using the NR econ software (version 1.6). The subchondral bone analysis was performed using the CT software (version 1.13). As reported in previous studies,^[^ ^22^ ^]^ one cubic region of interest (0.5  ×  0.5  ×  0.5 mm) beneath the center top of the condyle was measured for the parameters, including percent bone volume (BV/TV), bone surface/volume ratio (BS/BV), bone mineral density (BMD), trabecular number (Tb.N), and trabecular separation (Tb.Sp).

### 2.11 Histological, Immunohistochemical, and Immunofluorescence Staining of Condylar Tissue

The other condyle from each rat was decalcified in 10% ethylenediamine tetraacetic acid (pH = 7.4) for 4 weeks. Then, the tissue was embedded in paraffin, and the embedding direction was strictly controlled to ensure that the masseter muscle was embedded parallel to the surface of the embedding box. The paraffin blocks were sliced into 6 µm sections. The sections were dewaxed with xylene and rehydrated with a graded ethanol series.

Slices around the maximum cross‐section of the condyle were selected and stained using hematoxylin‐eosin (HE), and Safranin O‐fast green (Solarbio). The slides were photographed under a microscope (Leica DM4000B) for further analysis. Based on the HE staining, cartilage thickness was measured according to the previous study.^[^ ^23^ ^]^ Condylar cartilage destruction was scored according to the Osteoarthritis Research Society International (OARSI) grading system.^[^ ^24^ ^]^

Immunohistochemical (IHC) staining was performed with antibodies against MMP13 (1:100; ab39012; Abcam) and MMP3 (1:100; ab52915; Abcam). Antigen retrieval was performed by treating the sections in 0.05% trypsin (pH = 7.8) at 37 °C for 30 min; the reaction was blocked with 3% BSA at 25 °C for 30 min, and the sections were incubated with primary antibodies at 4 °C overnight. Then, the sections were incubated with an HRP‐labeled goat anti‐rabbit secondary antibody (1:200; GB23303; Servicebio) at 25 °C for 50 min. Next, the sections were stained with the 3,3′‐diaminobenzidine substrate and hematoxylin. Finally, the slices were dehydrated and mounted. The slides were photographed under a microscope (Leica DM4000B), and the results were quantified involving the whole cartilage layer using the *Trainable Weka Segmentation* plugin in the *ImageJ* software (National Institutes of Health) as previously reported.^[^ ^25^ ^]^

Immunofluorescence staining was performed with antibodies against VIM (1:200; ab92547; Abcam), ORM1 (1:100; MA5‐32836; Invitrogen), Phospho‐ERK (1:200; 4370; Cell Signaling Technology), Phospho‐JNK (1:200; 4668; Cell Signaling Technology), and Phospho‐p38 (1:200; 4511; Cell Signaling Technology). Antigen retrieval was performed by treating the sections with 0.05% trypsin (pH = 7.8) at 37 °C for 30 min; the reaction was blocked with 3% BSA at 25 °C for 30 min, and the sections were incubated with primary antibodies at 4 °C overnight. Then, the sections were incubated with the goat anti‐mouse IgG H&L (Alexa Fluor 488; 1:400; ab150113; Abcam) and/or goat anti‐rabbit IgG H&L (Alexa Fluor 594; 1:400; ab150080; Abcam) at 25 °C for 50 min. Afterward, the sections were incubated with DAPI (Servicebio) at 25 °C for 10 min and tissue autofluorescence quencher (Servicebio) at 25 °C for 5 min, and then mounted. The slides were photographed under a microscope (Leica DM4000B), and the results were quantified involving the whole cartilage layer using the *ImageJ* software. In particular, the *Colocalization‐Finder* plugin in *ImageJ* software was used to evaluate the immunocolocalization levels of ORM1 and VIM, which were shown using the Pearson's coefficient and Overlap coefficient.

### 2.12 RNA Isolation and Bulk RNA Sequencing

C28/I2 cells were seeded in six‐well plates and incubated with equivalent phosphate‐buffered saline (PBS) as the natural control (NC, n = 3), 10 ng mL^−1^ Interleukin‐1β (IL‐1β, Peprotech, n = 3), or IL‐1β in the presence of 200 µg mL^−1^ ORM1 protein (n = 3) for 24 h. For bulk RNA sequencing, total RNA was extracted using TRIzol Reagent (Thermo). The NanoDrop 2000 spectrophotometer (Thermo) was used to evaluate RNA purity and quantity. RNA integrity was evaluated by the Agilent 2100 Bioanalyzer (Agilent Technologies). The TruSeq Stranded mRNA LT Sample Prep Kit (Illumina) was used to construct the sequencing libraries. Transcriptome sequencing and analysis were performed by OE Biotech Co., Ltd. (Shanghai, China).

### 2.13 Bulk RNA Sequencing Analysis

Trimmomatic was used to process the raw data (raw reads). Clean reads were obtained by removing poly‐N‐containing reads and low‐quality reads. Then, the clean reads were mapped to the human genome (GRCh38) by *HISAT2*.^[^ ^26^ ^]^ The fragments per kilobase million (FPKM) and transcripts per million (TPM) of each gene were obtained using *Cufflinks*, ^[^ ^27^ ^]^ and the read counts of each gene were obtained by HTSeq count.^[^ ^28^ ^]^ Principal component analysis (PCA) was performed with TPM values using *prompt* in the R package *stats*. The *limma* package was used for differential expression analysis.^[^ ^29^ ^]^ An adjusted P value (false discovery rate [FDR] suggested by Benjamini and Hochberg) of <0.05 and |log2foldchange| of >0.38 was set as the threshold to determine significant differential expression. The DEGs were further analyzed by pathway enrichment analysis.

### 2.14 Reverse‐Transcription Quantitative Polymerase Chain Reaction

Cells were collected for reverse‐transcription quantitative polymerase chain reaction (RT‐qPCR) analysis. Total RNA was extracted using TRIzol reagent. Complementary DNA was synthesized using an RT reagent kit (Yeason, China), and RT‐qPCR was performed using SYBR Green Master Mix (Yeason, China) to determine the RNA expression. According to the manufacturer's protocol, PCR was performed under the following conditions: an initial denaturation step at 95 °C for 2 min, 40 cycles of amplification (95 °C for 10 s, and 60 °C for 30 s), and a final extension step at the melting curve stage. The expression levels were calculated using the 2^‐ΔΔCt^ method and normalized to glyceraldehyde‐3‐phosphate dehydrogenase (GAPDH). The primers are shown in Table 2 (Supporting Information).

### 2.15 In Vitro Transfection with Plasmids and siRNAs

The plasmids overexpressing ORM1 (NCBI gene ID: 5004, ORM1–Flag), 52 – 167 amino acid truncation of ORM1 [ORM1(p.52to167) –Flag], the truncation of ORM1 with the deletion of 52 – 167 amino acids [ORM1(p.52to167del) –Flag], and VIM (NCBI gene ID: 7431, VIM–Myc) were obtained from Genomeditech (China). Plasmid transfection was performed using Lipofectamine 3000 (Thermo). For a single well in a six‐well plate, 5 µL Lipofectamine 3000 or 1 µg plasmids with 5 µL p3000 were added to 125 µL OPTI‐MEM(R) I reduced serum medium (Gibco), and the plate was placed at 25 °C for 5 min. Then, the solutions were mixed thoroughly and placed at 25 °C for 20 min. The mixture was added to cells cultured with 2 mL medium in the six‐well plates.

The ORM1 siRNA, VIM siRNA and NC siRNA were obtained from GenePharma (Shanghai, China); the siRNA sequences are listed in Table S3 (Supporting Information). Cell transfection with siRNA was performed using the transfection reagent GP‐transfect‐mate. For a single well in a six‐well plate, 150 pmol siRNA or 8 µL GP‐transfect‐mate were respectively added to 200 µL OPTI‐MEM(R) I medium and placed at 25 °C for 5 min. Then, the solutions were mixed thoroughly and placed at 25 °C for 20 min. The mixture was added to cells cultured with 2 mL medium in the six‐well plates. To explore the changes in phosphorylation of ERK, JNK, and p38, cells were collected for various experiments at 12 h after transfection. To explore the changes in MMP3 and MMP13, cells were collected for various experiments at 24 h after transfection.

### 2.16 Western Blot

Cells were harvested and lysed, and equal quantities of protein were separated using sodium dodecyl sulfate polyacrylamide gel electrophoresis (SDS‐PAGE) gel and transferred to a nitrocellulose membrane (Pall). The membrane was then blocked with 5% nonfat dry milk for 1 h and incubated overnight with primary antibodies. After the membrane was washed thrice with TBS supplemented with 0.1% Tween‐20 (TBST), it was incubated with the secondary antibody. Detection was performed using Immobilon ECL Ultra Western HRP Substrate (Millipore). The antibodies are shown in Table S4 (Supporting Information).

### 2.17 Immunofluorescence in Cells

Cells with various treatments were cultured in confocal dishes (Cellvis). After administration, cells were fixed in 4% polyformalin for 15 min, incubated with 0.25% Triton X‐100 (Sigma–Aldrich) for 10 min, and blocked with 3% BSA for 1 h. Then, cells were incubated with the antibodies aainst MMP13 (1:100; ab39012; Abcam), MMP3 (1:100; ab52915; Abcam), VIM (1:200; ab92547; Abcam) or VIM (1:200) plus ORM1 (1:100; MA5‐32836; Invitrogen) at 4 °C overnight. Next day, cells were incubated with goat anti‐rabbit IgG H&L (Alexa Fluor 488; 1:400; ab150077; Abcam), or goat anti‐mouse IgG H&L (Alexa Fluor 488; 1:400; ab150113; Abcam) plus goat anti‐rabbit IgG H&L (Alexa Fluor 594; 1:400; ab150080; Abcam) at 37 °C for 1 h. Finally, cells were mounted in antifade mounting medium with DAPI (Biosharp) for fluorescence. For immunocolocalization assays in C28/I2 cells, cells were photographed using a confocal laser scanning microscope (Leica). Others were photographed by another microscope (Leica DFC7000T). Fluorescence intensity was quantified using *ImageJ* software. In particular, the *Colocalization‐Finder* plugin in *ImageJ* software was used to evaluate the immunocolocalization levels, which were shown using the Pearson's coefficient and Overlap coefficient.

### 2.18 Co‐Immunoprecipitation (coIP)

After transfection with ORM1‐Flag plasmid for 24 h, IP Lysis Buffer (Beyotime Biotechnology) was used to lyse cells. Then, the primary antibody was added to the lysate overnight at 4 °C. Then, the lysate was incubated with 30 µl of Protein G‐Agarose (Sigma–Aldrich) for 2 h. After being washed with the lysis buffer three times, the beads were mixed with loading buffer (Takara) and boiled for 20 min at 60 °C. The samples were used for coomassie western blot or brilliant blue staining.

### 2.19 Coomassie Brilliant Blue Staining in SDS‐PAGE Gels and LC‐MS/MS Characterization

The proteins obtained from coIP were separated by electrophoresis on SDS‐PAGE gels. Then, the gels were stained by coomassie brilliant blue R 250 (Sigma), and destained by washing in a destaining solution thrice for 1 h each time. Gels containing proteins with different shades of stain in the control and experimental lanes were cut, and the proteins were digested with trypsin. The peptides were separated by LC‐MS/MS using ESI‐QUAD‐TOF (Boyuan Biotech), and were identified using the NCBI protein database. Based on the structure of ORM1 and VIM,^[^ ^30^ ^]^ docking between ORM1 and VIM was predicted by ZDOCK.

### 2.20 The Inhibition of MAPK Pathway

Cells were seeded in six‐well plates and transfected with Myc or VIM–Myc plasmids for 24 h. Then, SCH772984 (Selleckchem), SP600125 (Selleckchem), and SB203580 (Selleckchem) were respectively used to inhibit ERK, JNK, and p38 at the concentration of 10 µmol L^−1^. The expression levels of MMP13 and MMP3 were detected after 24 h using western blot.

### 2.21 Statistical Analysis

The data were analyzed using R version 4.1.0 (www.r‐project.org)^[^ ^19^ ^]^ or GraphPad Prism 8.0. Continuous variables were shown as mean ± standard deviation (SD). Differences between two groups were analyzed using unpaired Student's t test (two‐tailed). For multiple group comparisons, one‐way analysis of variance (ANOVA) followed by Tukey's post‐hoc test for multiple comparisons was performed. A P value <0.05 was considered to indicate a significant difference. All experiments were independently repeated at least three times to ensure reproducibility.

## 3 Results

### 3.1 Downregulation of ORM1 in Patients with Temporomandibular Joint Osteoarthritis

A total of 63 adolescent patients with ADD‐related TMJOA were included in this study, including patients with mild (*n* = 21), moderate (*n* = 27), and severe (*n* = 15) TMJOA (Figure 1A). SF samples were collected from these patients during the disc repositioning surgery (Figure 1B) and examined using proteomic analysis (Figure 1C). There was no significant difference in the number of identified proteins in the SF samples among the three groups (Figure S1A, Supporting Information). In the 63 samples, 1548 proteins were identified by data‐independent acquisition mass spectrometry; their subcellular locations were mainly cytoplasm (43.5%), cell membrane (34.1%), and secreted (27.0%; Figure S1B). The cellular component terms associated with these proteins included vesicle lumen, secretory granule lumen, cytoplasmic vesicle lumen, and collagen‐containing extracellular matrix (Figure S1C, Supporting Information). The biological processes associated with these proteins included coagulation, wound healing, and regulation of body fluid levels (Figure S1D, Supporting Information). PCA showed distinction among the three patient groups (Figure 1D). Following the analysis of the differentially identified proteins among the three groups, ORM1 attracted attention, as its levels showed a significant downward trend with disease progression (Figure 1E,F). Moreover, patients with pain showed significantly lower ORM1 levels than those without pain (Figure 1G), and no significant difference was observed among patients with different MIO (Figure 1H) or between patients with or without bruxism (Figure 1I). Further ELISA assay confirmed the downward trend with disease progression in SF samples (Figure 1J). Besides, to explore whether the proteins in SF are associated with the extent of pain and MIO, WGCNA was performed, which showed that the module Yellow (105 proteins) was significantly positively correlated with the visual analogue scale (VAS) score of pain (Figure S1E, Supporting Information). The interaction network showed that the proteins in the module Yellow were enriched in terms such as neutrophil degranulation and supramolecular fiber organization (Figure S1F, Supporting Information).

### 3.2 ORM1 Injection Attenuates the Progression of Temporomandibular Joint Osteoarthritis in Unilateral Anterior Crossbite Rats

**Figure 2:**
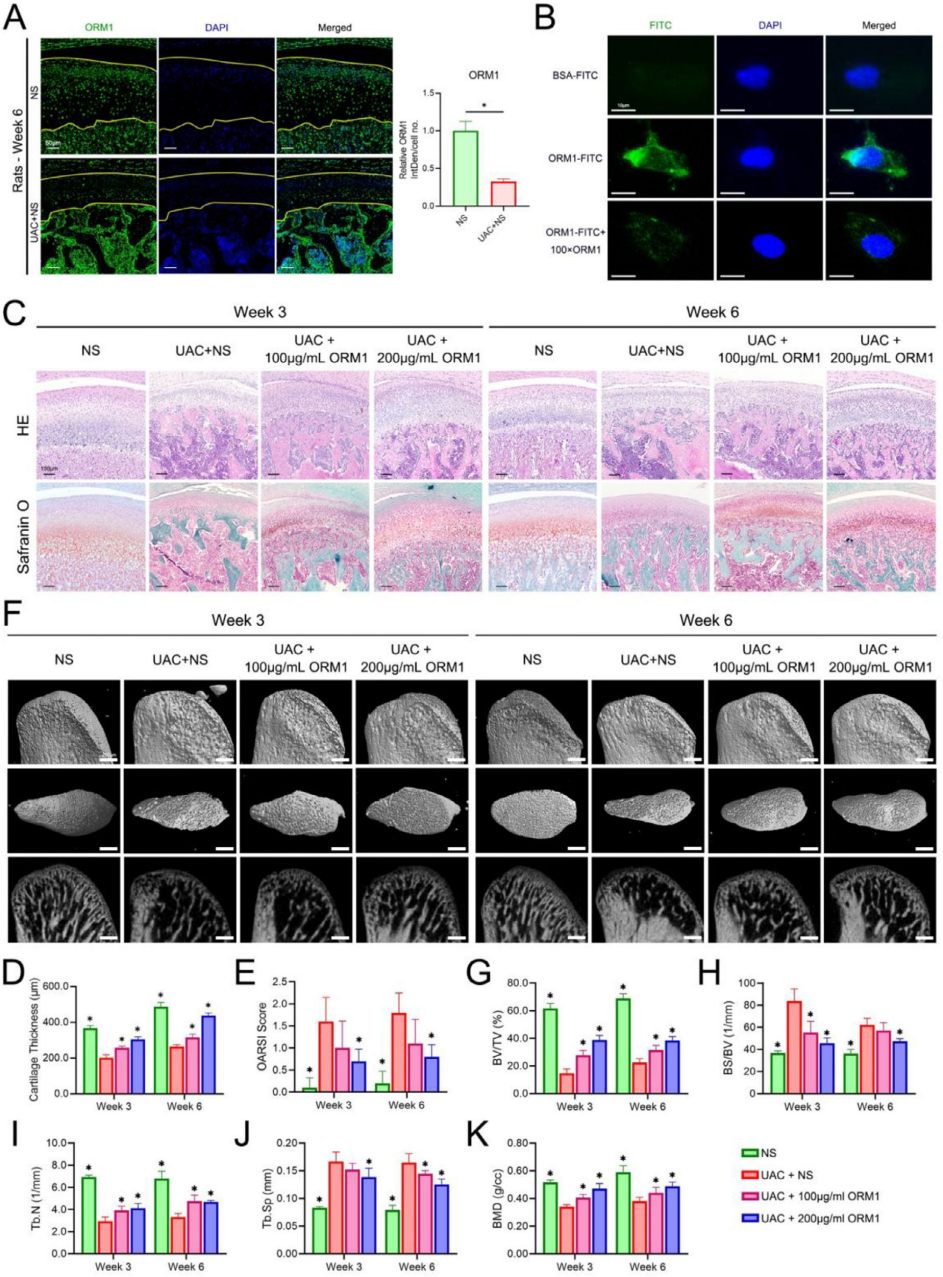
Histopathological and microcomputed tomography (micro‐CT) analysis showing the effect of ORM1 on maintaining cartilage homeostasis in rats with TMJOA. A) Immunofluorescence analysis of ORM1 in the condylar cartilage of rats with or without TMJOA. Data are expressed as mean ± SD and analyzed using unpaired Student's t test (two‐tailed), n = 3, *p < 0.05. B) Subcellular localization of exogenous ORM1 conjugated with fluorescein isothiocyanate (FITC). C) Hematoxylin–eosin and Safranin O‐fast green staining of the condylar cartilage of rats in various treatment groups. D) Thickness of the condylar cartilage of rats in various treatment groups based on the Hematoxylin–eosin staining. Data is expressed as mean ± SD and analyzed using one‐way ANOVA followed by Tukey's post‐hoc test, n = 5, *p < 0.05 compared with the UAC+NS group. E) Osteoarthritis Research Society International (OARSI) scores of the condylar cartilage of rats in various treatment groups based on the Safranin O‐fast green staining. Data is expressed as mean ± SD and analyzed using one‐way ANOVA followed by Tukey's post‐hoc test, n = 5, *p < 0.05 compared with the UAC+NS group. F) Micro‐CT images of condylar tissue samples from various treatment groups. Comparison of percent bone volume (BV/TV) (G), bone surface/volume ratio (BS/BV) (H), trabecular number (Tb.N) (I), trabecular separation (Tb.Sp) (J), and bone mineral density (BMD) K) among various treatment groups. NS, normal saline; UAC, unilateral anterior crossbite. Data is expressed as mean ± SD and analyzed using one‐way ANOVA followed by Tukey's post‐hoc test, n = 5, *p < 0.05 compared with the UAC+NS group.

ORM1 expression was observed in the condylar cartilage in both control and UAC rats. It showed that ORM1 expression in the condylar cartilage at 6 weeks after the construction of UAC was lower than that in the control group, and ORM1 was mainly expressed in the deep zone (hypertrophic layer) of condylar cartilage in the UAC rats (**Figure** **2**A). The therapeutic effect of ORM1 in TMJOA was further explored in vivo. First, a cell proliferation assay was used to determine that 200 µg mL^−1^ of ORM1 was a suitable concentration to treat chondrocytes (Figure S2, Supporting Information). Then, it showed that ORM1 can be taken up by cells through endocytosis (Figure 2B). Thus, after the construction of UAC at week 0, 50 µL NS or ORM1 dissolved in NS (100 or 200 µg mL^−1^) was injected into the TMJ capsules of UAC rats from week 1 to week 2 or week 1 to week 5. Then, the rats were euthanized at week 3 or week 6, respectively, and the condyles were harvested (Figure S3A, Supporting Information). There was no significant difference in body weight among the three groups of UAC rats injected with either NS or ORM1 (Figure S3B, Supporting Information). The condyles were collected for pathological analyses (Figure 2C). HE staining showed the rough surface and ossified regions in UAC groups, especially in rats treated for 6 weeks. ORM1 injection recovered the decrease in cartilage thickness in the UAC groups (Figure 2D). In comparison with the UAC groups, OARSI scores of the UAC + 200 µg mL^−1^ ORM1 group were significantly lower than those of the UAC+NS group (Figure 2E). Subchondral bone remodeling was assessed using micro‐CT. In both week 3 and week 6, the NS group showed the densest trabeculae, whereas the UAC group showed clear hollow trabeculae. Trabecular density recovered significantly in the two UAC + ORM1 groups (Figure 2F). Such changes were validated by further analyses. The UAC group showed lower BV/TV, Tb.N and BMD, and higher BS/BV and Tb.Sp than the NS group. However, treatment with ORM1 reversed the changes, with ORM1‐treated groups showing higher BV/TV, Tb.N and BMD, and lower BS/BV and Tb.Sp than the NS‐treated UAC group (Figure 2G–K).

### 3.3 ORM1 Participated in Regulating Cartilage Homeostasis by Inhibiting the Expression of MMP13 and MMP3

**Figure 3:**
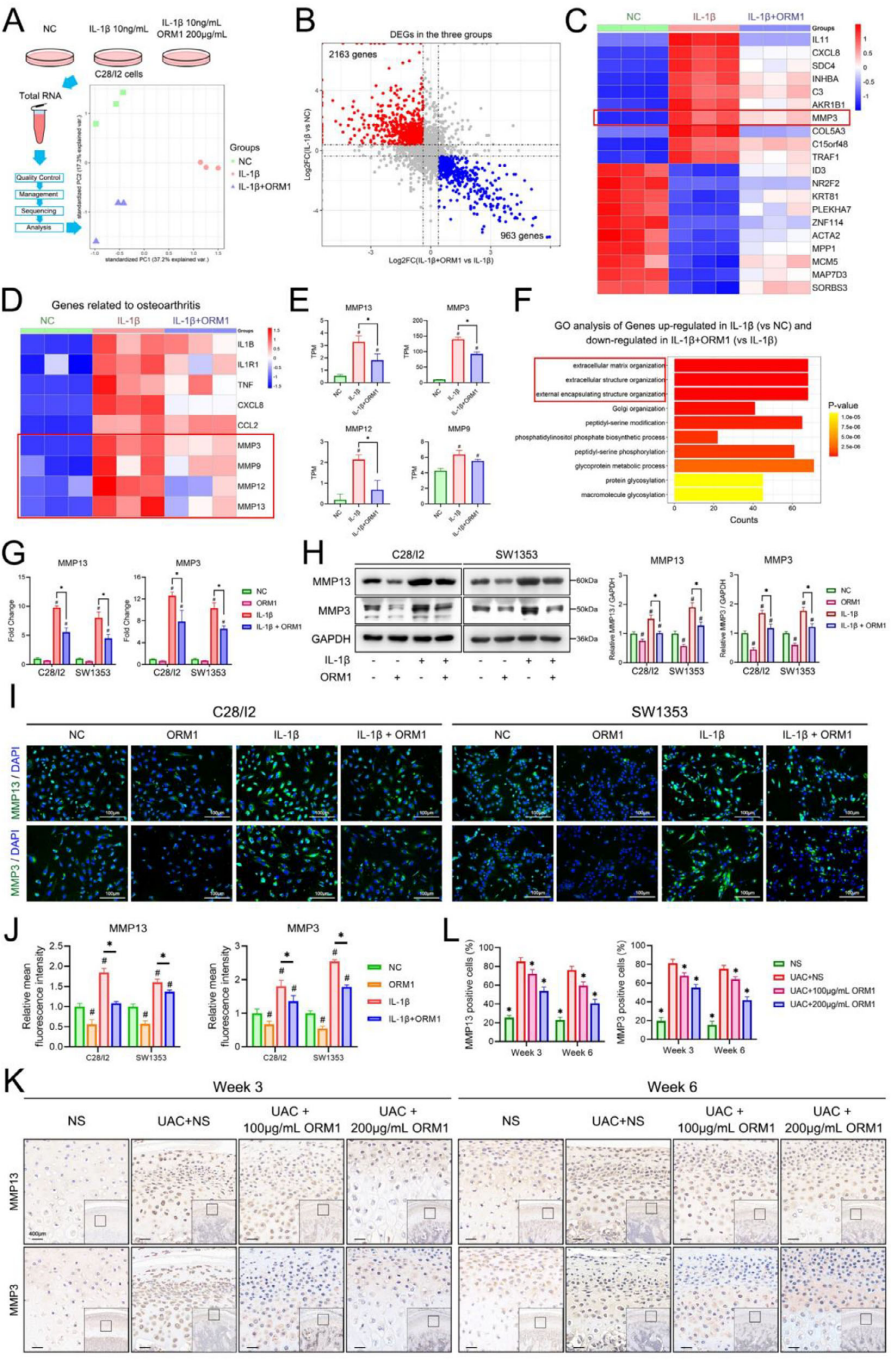
Bulk RNA sequencing in C28/I2 cells treated with ORM1, and the inhibitory effect of ORM1 on MMP13 and MMP3. A) Administration on C28/I2 cells and the principal component analysis; B) Volcano map showing the differentially expressed genes (DEGs) of interest; C) Top 20 DEGs rescued after ORM1 treatment; D) Expression change of genes related to osteoarthritis in various treatment groups; E) The transcripts per million (TPM) of MMP13, MMP3, MMP12, and MMP9 in the three groups. Data is expressed as mean ± SD and analyzed using one‐way ANOVA followed by Tukey's post‐hoc test, n = 3, ^#^ *p* < 0.05 compared with the NC group, *p < 0.05. F) GO analysis of DEGs that were upregulated after IL‐1β treatment and downregulated after ORM1 treatment. G) mRNA expression level of MMP13 and MMP3 in both natural and inflammation environments in C28/I2 and SW1353 cells after treatment with ORM1 protein. Data is expressed as mean ± SD and analyzed using one‐way ANOVA followed by Tukey's post‐hoc test, n = 3, ^#^ *p* < 0.05 compared with the NC group, *p < 0.05. H) Western blot showing the protein level of MMP13 and MMP3 in both natural and inflammatory environments in C28/I2 and SW1353 cells after treatment with ORM1 protein. Data is expressed as mean ± SD and analyzed using one‐way ANOVA followed by Tukey's post‐hoc test, n = 3, ^#^ *p* < 0.05 compared with the natural control (NC) group, *p < 0.05. I, J) Immunofluorescence analysis showing the effect of ORM1 on MMP13 and MMP3 in vitro. Data is expressed as mean ± SD and analyzed using one‐way ANOVA followed by Tukey's post‐hoc test, n = 3, ^#^ *p* < 0.05 compared with the NC group, *p < 0.05. K) Immunohistochemical staining of MMP13 and MMP3 in the condylar cartilage of rats among various treatment groups. L) Comparison of MMP13‐ and MMP3‐ positive cells in rats among various treatment groups. Data is expressed as mean ± SD and analyzed using one‐way ANOVA followed by Tukey's post‐hoc test, n = 5, *p < 0.05 compared with UAC+NS group.

C28/I2 cells were treated with PBS, 10 ng/mL IL‐1β, or IL‐1β + 200 µg mL^−1^ ORM1 for 24 h. Then, the cells were collected for RNA sequencing. PCA showed a clear separation among the three groups (**Figure** **3**A). Further analysis found 2163 genes upregulated after IL‐1β treatment and downregulated after ORM1 treatment. Additionally, 963 genes were downregulated after IL‐1β treatment and upregulated after ORM1 treatment (Figure 3B). The top 20 genes whose expression was rescued after ORM1 treatment are shown in Figure 3C. Moreover, genes associated with osteoarthritis were of interest (Figure 3D). The expression of MMP13, MMP3, and MMP12 was significantly rescued by ORM1 treatment (Figure 3E). Except CXCL5 and CXCL8, inflammation‐related genes showed no significant change in expression after ORM1 treatment, including IL1B, IL1R1, TNF, CXCL10, CCL2, CCL4, and CCL7 (Figure S4A, Supporting Information). Genes that were upregulated after IL‐1β treatment and downregulated after ORM1 treatment were enriched in GO terms closely associated with extracellular matrix organization, which includes the MMP13 and MMP3 genes (Figure 3F). KEGG pathway analysis indicated that these genes were enriched in ECM–receptor interaction, which includes the MMP13 and MMP3 genes (Figure S4B, Supporting Information). Further analysis with Metascape indicated that these genes were involved in pathways such as cellular response to cytokine stimulus, signaling by TGFB family members, and regulation of cellular response to stress (Figure S4C, Supporting Information). Notably, MMP3 was involved in the top two enriched pathways in the Metascape analysis: the NABA matrisome‐associated pathway (Figure S4D, Supporting Information) and the extracellular matrix organization pathway (Figure S4E, Supporting Information). Thus, MMP3 was remarkable. Genes that were downregulated after IL‐1β treatment and upregulated after ORM1 treatment were enriched in GO terms closely associated with RNA splicing (Figure S4F, Supporting Information) and KEGG pathways associated with spliceosomes and phagosomes (Figure S4G, Supporting Information).

Owing to the key role of MMP13 and MMP3 in osteoarthritis, these two genes were of significant interest. In both C28/I2 and SW1353 cells, MMP13 and MMP3 mRNA expression decreased significantly in inflammatory environments after treatment with ORM1 (Figure 3G) or ORM1–Flag plasmid (Figure S5A, Supporting Information). At the protein level, the expression results were consistent (Figure 3H; Figure S5B, Supporting Information). Immunofluorescence analysis showed that in both types of cells, treatment with ORM1 significantly decreased MMP13 and MMP3 levels (Figure 3I,J). The effect of ORM1 on the MMPs was further confirmed in vivo. Immunohistochemical (IHC) staining showed that MMP13 and MMP3 were expressed in all layers of condylar cartilage in the UAC rats, and only the chondrocytes in the mature layer and some part of the hypertrophic layer expressed MMP13 and MMP3 in the rats treated with 200 µg mL^−1^ ORM1 (Figure 3K). Further analysis showed that the proportion of cells expressing MMP13 and MMP3 was higher in the UAC groups than in the control group, but fewer condylar cells expressed these two markers in the ORM1 treatment groups (Figure 3L).

### 3.4 Identification of Interaction between ORM1 and VIM

**Figure 4:**
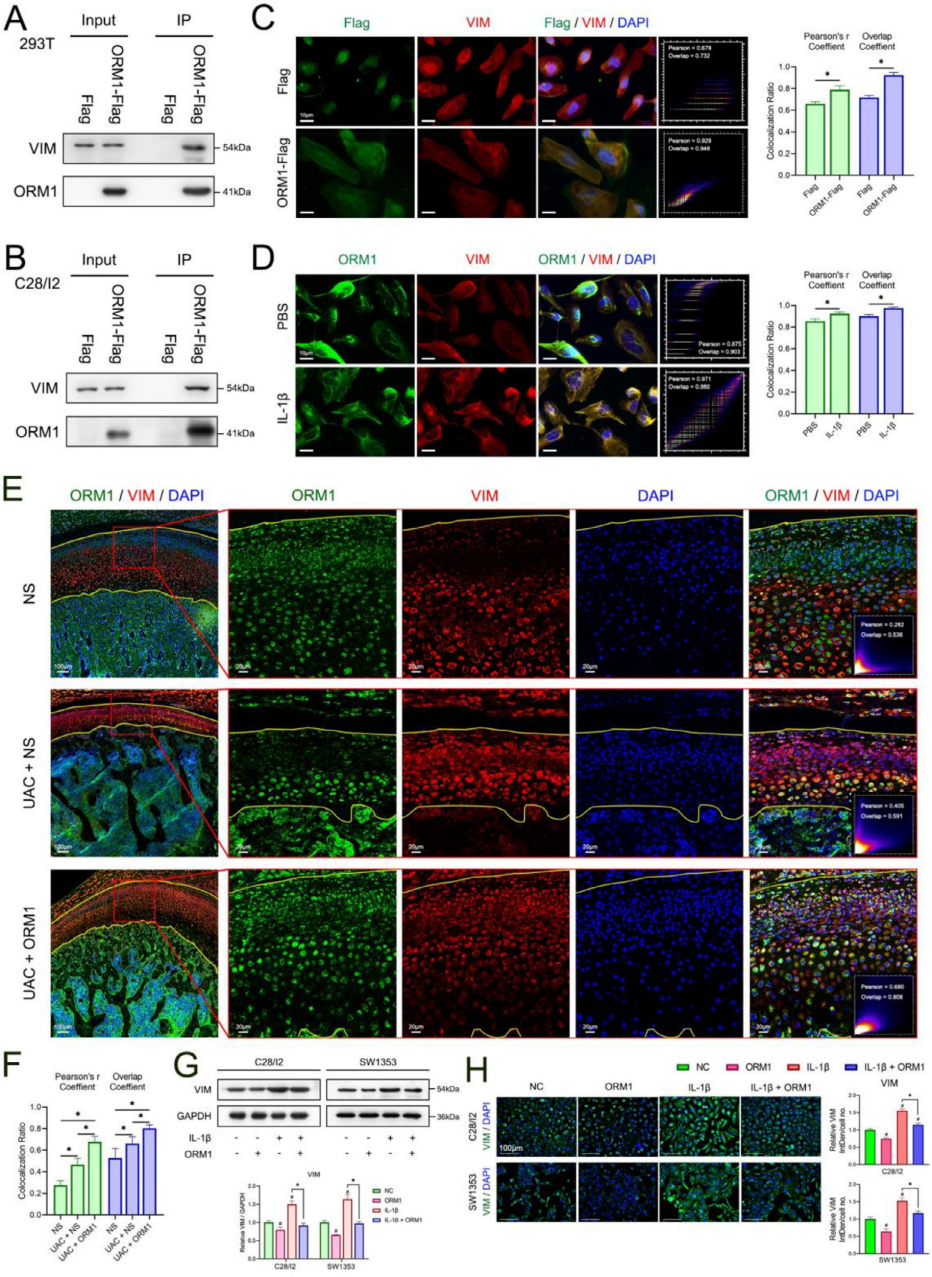
Identification of the interaction between ORM1 and vimentin (VIM). Co‐immunoprecipitation (CoIP) was used to detect binding between ORM1 and VIM in HEK‐293T (A) and C28/I2 cells (B). An immunocolocalization assay was used to examine the colocalization of VIM and exogenous ORM1 (C) or endogenous ORM1 (D) in C28/I2 cells. Data are expressed as mean ± SD and analyzed using unpaired Student's t test (two‐tailed), n = 3, *p < 0.05. Immunocolocalization (E) and quantification (F) of ORM1 and VIM in the condylar cartilage of rats in the three treatment groups, with the yellow lines showing the articular surface and the interface between bone and cartilage. Data is expressed as mean ± SD and analyzed using one‐way ANOVA followed by Tukey's post‐hoc test, n = 5, *p < 0.05. Western blot (G) and immunofluorescence (H) showing the protein level of VIM in both natural and inflammation environments in C28/I2 and SW1353 cells after treatment with ORM1 protein. Data is expressed as mean ± SD and analyzed using one‐way ANOVA followed by Tukey's post‐hoc test, n = 3, *p < 0.05, ^#^ *p* < 0.05 compared with the NC group.

To further explore the mechanism of the therapeutic effect of ORM1 in TMJOA, the coIP assay was performed, and the proteins were stained with coomassie brilliant blue after separating in SDS‐PAGE gels. The results showed that proteins around 50 kDa were different between the IgG and IP lane. Then, LC‐MS/MS identified VIM as the sole co‐immunoprecipitated protein of ORM1 at around 50 kDa (Figure S6A, Supporting Information). Docking between ORM1 and VIM was predicted by ZDOCK (Figure S6B, Supporting Information). The binding was confirmed by performing a coIP assay in HEK‐293T cells (**Figure** **4**A) and C28/I2 cells (Figure 4B). Immunocolocalization of exogenous ORM1 and VIM confirmed the binding in C28/I2 cells (Figure 4C). Moreover, endogenous ORM1 and VIM showed more overlap in the inflammation environment (Figure 4D). To further confirm the binding, the condylar cartilage from rats was used to perform the immunocolocalization assay. It showed that ORM1 was localized in all the layers of condylar cartilage in the NS group and UAC+ORM1 group, but was only localized in the hypertrophic layer of condylar cartilage in the UAC rats. VIM was localized in all the layers of condylar cartilage in the UAC and UAC+ORM1 groups, and it showed that chondrocytes in the hypertrophic layer of condylar cartilage in the UAC+ORM1 group expressed less VIM than the UAC group. The colocalization of ORM1 and VIM in the condylar cartilage was more significant in the UAC group than in the NS group and even more significant in the ORM1 treatment group (Figure 4E,F). To explore the effect of the binding on the disease, the expression of VIM was detected. In both C28/I2 and SW1353 cells, western blot (Figure 4G) and immunofluorescence (Figure 4H) showed that treatment with ORM1 protein significantly decreased the VIM protein level, especially in the inflammation environment.

### 3.5 ORM1 Downregulated VIM and MMPs Expression and Inhibited MAPK Pathway

**Figure 5:**
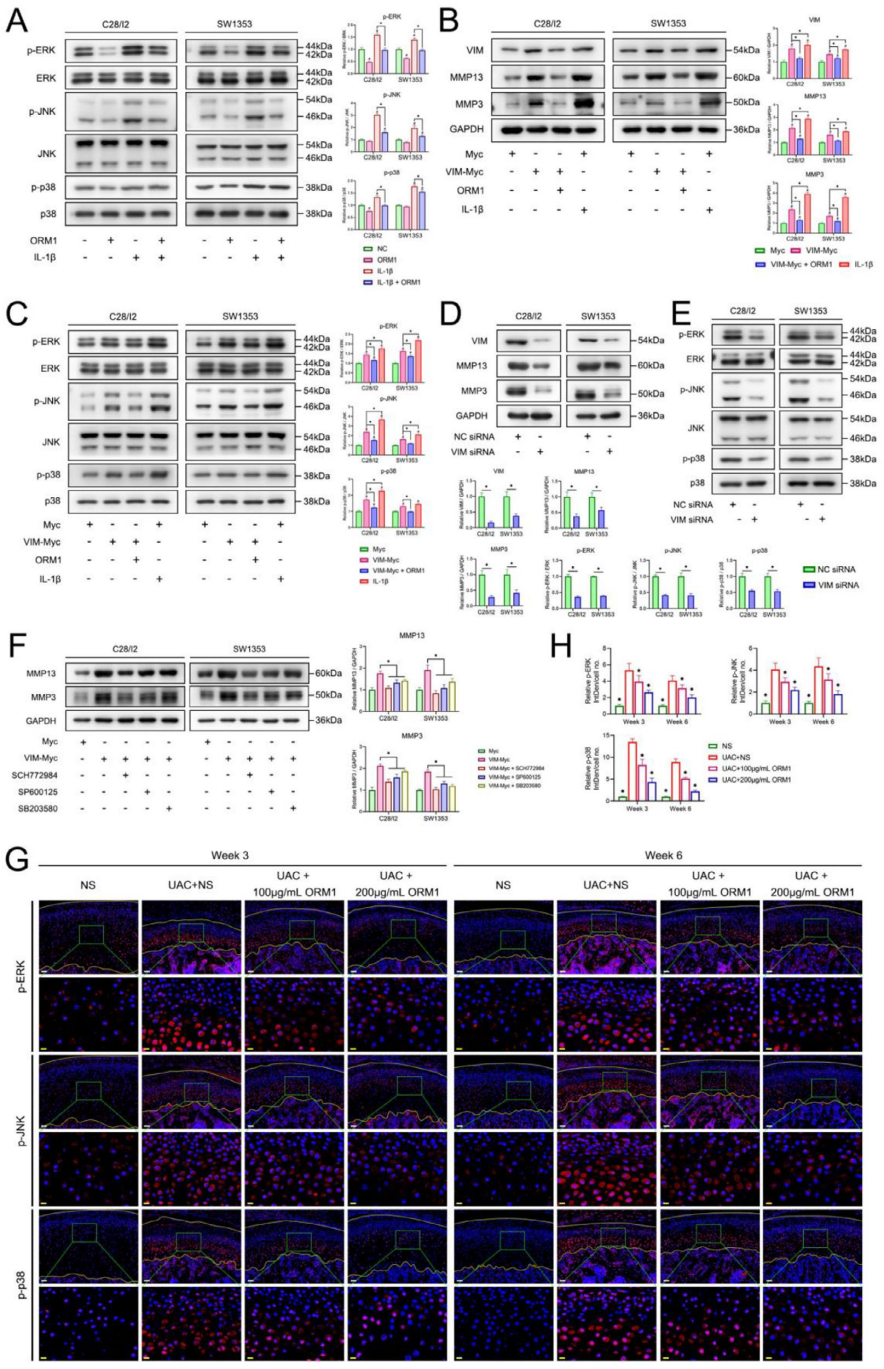
Inhibitory effect of ORM1 on VIM/MAPK/MMP signaling pathway. A) Effect of ORM1 treatment on the phosphorylation of ERK, JNK, and p38 in C28/I2 and SW1353 cells was detected using western blot. Data is expressed as mean ± SD and analyzed using one‐way ANOVA followed by Tukey's post‐hoc test, n = 3, *p < 0.05, ^#^ *p* < 0.05 compared with the NC group. Expression of MMP13 and MMP3 (B), and the phosphorylation of extracellular signal‐regulated kinase (ERK), c‐Jun N‐terminal kinase (JNK), and p38 mitogen‐activated protein kinase (p38) (C) after overexpression of VIM with or without ORM1 treatment in C28/I2 and SW1353 cells. Data is expressed as mean ± SD and analyzed using one‐way ANOVA followed by Tukey's post‐hoc test, n = 3, *p < 0.05, ^#^ *p* < 0.05 compared with the Myc group. Expression of MMP13 and MMP3 (D), and the phosphorylation of ERK, JNK, and p38 (E) after knockdown of VIM in C28/I2 and SW1353 cells. Data is expressed as mean ± SD and analyzed using unpaired Student's t test (two‐tailed), n = 3, *p < 0.05, ^#^ *p* < 0.05 compared with the NC siRNA group. F) Expression of MMP13 and MMP3 after overexpression of VIM with or without inhibition of ERK, JNK, and p38 in C28/I2 and SW1353 cells. Data is expressed as mean ± SD and analyzed using one‐way ANOVA followed by Tukey's post‐hoc test, n = 3, *p < 0.05. G) The phosphorylation levels of ERK, JNK, and p38 in condylar cartilage of rats were detected by immunofluorescence, with the yellow lines showing the articular surface and the interface between bone and cartilage. H) Quantification of the phosphorylation levels of ERK, JNK, and p38 in condylar cartilage of rats based on the immunofluorescence. Data is expressed as mean ± SD and analyzed using one‐way ANOVA followed by Tukey's post‐hoc test, n = 5, *p < 0.05 compared with the UAC+NS group.

Several previous studies have reported the effect of VIM on MAPK pathway.^[^ ^31^ ^]^ Further assay showed that the phosphorylation of ERK, JNK, and p38 was downregulated after ORM1 treatment in both the natural and inflammation environments (**Figure** **5**A). In addition, the inhibition of ORM1 by siRNA upregulated the expression of VIM, MMP13, and MMP3 (Figure S7A, Supporting Information), and activated the phosphorylation of ERK, JNK, and p38 (Figure S7B, Supporting Information). VIM expression was upregulated by transfection with the VIM‐Myc plasmid. Cells treated with only the Myc plasmid or Myc plasmid plus IL‐1β were used as negative and positive control, respectively. Results showed that VIM overexpression upregulated MMP13 and MMP3 (Figure 5B), and promoted the phosphorylation of ERK, JNK, and p38 (Figure 5C), while ORM1 treatment can reverse the influence of VIM overexpression. Moreover, VIM knockdown downregulated MMP13 and MMP3 (Figure 5D), and inhibited the phosphorylation of ERK, JNK, and p38 (Figure 5E). When cells transfected with VIM‐Myc were treated with inhibitors of ERK (SCH772984), JNK (SP600125), or p38 (SB203580), the induction of MMP13 and MMP3 by VIM overexpression was significantly reduced (Figure 5F). The inhibition of MAPK pathway activation by ORM1 treatment was confirmed in vivo (Figure 5G,H).

### 3.6 The Effect of ORM1 Depends on Its Structure of 52 to 167 Amino Acids

**Figure 6:**
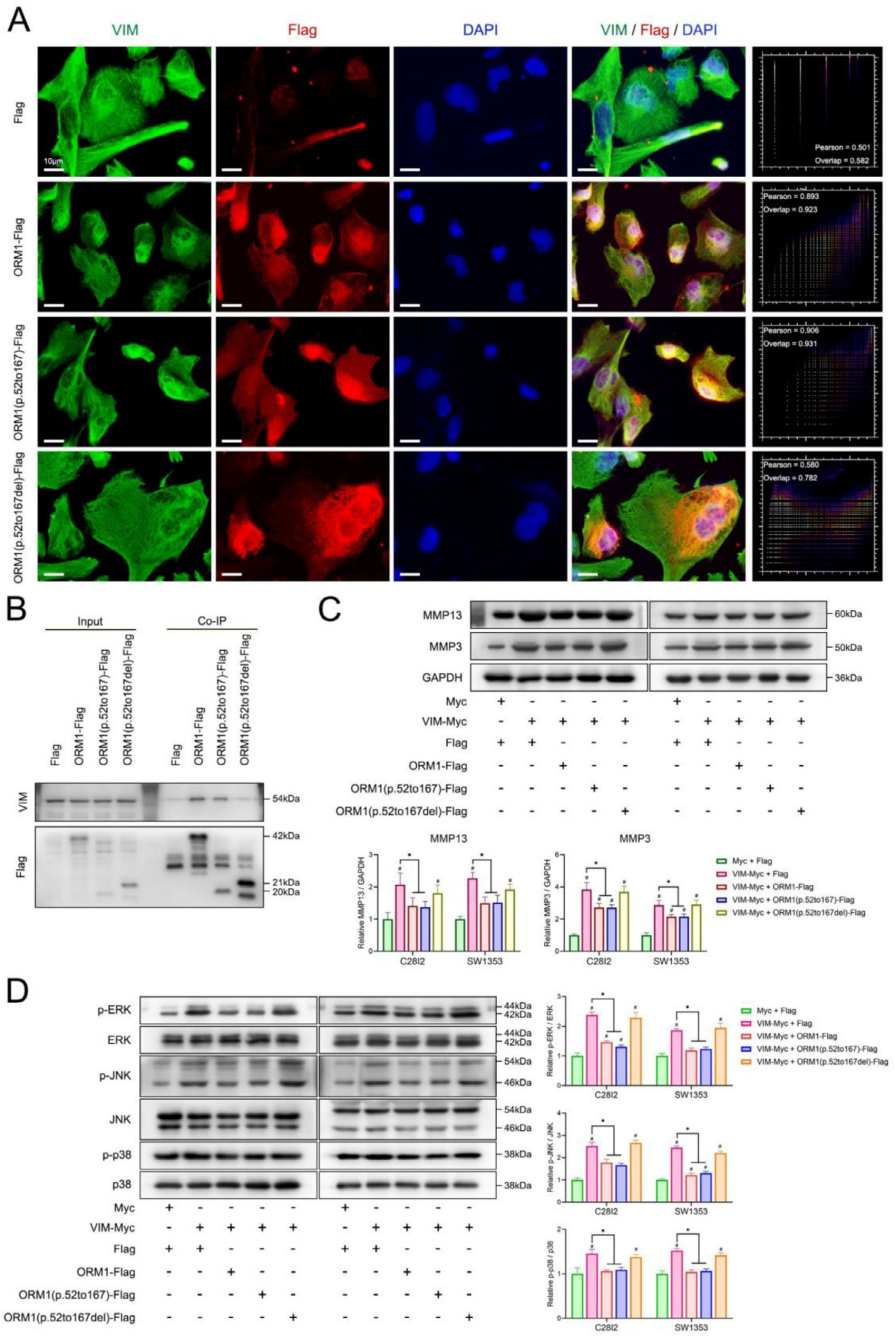
The role of ORM1‐VIM binding on the activities of MAPK pathway and the expression of MMPs. A) Immunocolocalization of ORM1 truncations and VIM in C28/I2 cells. B) CoIP of ORM1 truncations and VIM in C28/I2 cells. C) Expression of MMP13 and MMP3 after overexpression of VIM without or with the ORM1 truncations in C28/I2 and SW1353 cells. Data is expressed as mean ± SD and analyzed using one‐way ANOVA followed by Tukey's post‐hoc test, n = 3, *p < 0.05, ^#^ *p* < 0.05 compared with the Myc+Flag group. D) Phosphorylation of ERK, JNK, and p38 after overexpression of VIM without or with the ORM1 truncations in C28/I2 and SW1353 cells. Data is expressed as mean ± SD and analyzed using one‐way ANOVA followed by Tukey's post‐hoc test, n = 3, *p < 0.05, ^#^ *p* < 0.05 compared with the Myc+Flag group.

To further explore whether ORM1 suppresses the MAPK pathway by binding to VIM thereby suppressing its function, the effect of the mutation at ORM1‐VIM interfaces on the activities of MAPK pathway and the expression of MMPs was evaluated. Based on the docking of ORM1 and VIM predicted by ZDOCK (Figure S6B, Supporting Information), the alpha‐helix and beta‐sheet in the 52 to 167 amino acid structure of ORM1 may play an important role in the binding of ORM1 and VIM (Figure S8A, Supporting Information). Thus, the plasmids ORM1(p.52to167) (Figure S8B, Supporting Information) and ORM1(p.52to167del) were constructed. The immunocolocalization assay showed the high overlap of VIM and ORM1(p.52to167) truncation, while it showed no binding between VIM and ORM1(p.52to167del) truncation (**Figure** **6**A). The coIP assay confirmed the binding between VIM and ORM1(p.52to167) truncation (Figure 6B). Further assays showed that ORM1(p.52to167) truncation downregulated the MMPs expression and inhibited the phosphorylation of ERK, JNK, and p38, while ORM1(p.52to167del) truncation had no effect (Figure 6C,D).

### 3.7 Inhibition of VIM Improved the Progression of Temporomandibular Joint Osteoarthritis In vivo

**Figure 7:**
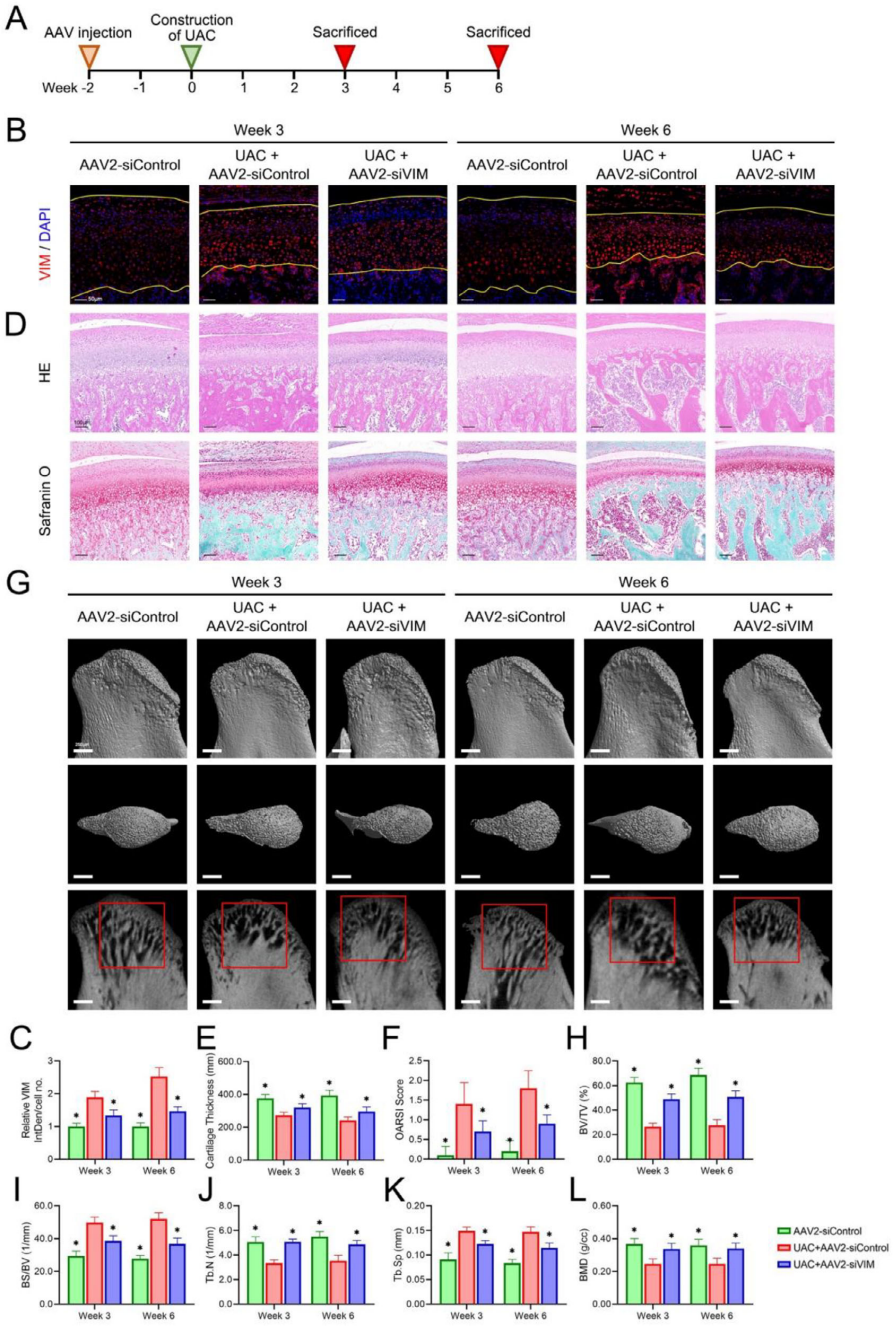
Effect of VIM inhibition in rats with TMJOA. A) Schematic diagram of the treatment process in rats. Immunofluorescence (B) and quantification (C) of VIM in condylar cartilage of rats, with the yellow lines showing the articular surface and the interface between bone and cartilage. Data is expressed as mean ± SD and analyzed using one‐way ANOVA followed by Tukey's post‐hoc test, n = 5, *p < 0.05 compared with the UAC+AAV2‐siControl group. D) Hematoxylin–eosin and Safranin O‐fast green staining of the condylar cartilage of rats in various treatment groups. E) Thickness of the condylar cartilage of rats in various treatment groups based on the Hematoxylin–eosin staining. Data is expressed as mean ± SD and analyzed using one‐way ANOVA followed by Tukey's post‐hoc test, n = 5, *p < 0.05 compared with the UAC+AAV2‐siControl group. F) OARSI scores of the condylar cartilage of rats in various treatment groups based on the Safranin O‐fast green staining. Data is expressed as mean ± SD and analyzed using one‐way ANOVA followed by Tukey's post‐hoc test, n = 5, *p < 0.05 compared with the UAC+AAV2‐siControl group. G) Micro‐CT images of condylar tissue samples from various treatment groups. Comparison of BV/TV (H), BS/BV (I), Tb.N (J), Tb.Sp (K), and BMD (L) among various treatment groups. Data is expressed as mean ± SD and analyzed using one‐way ANOVA followed by Tukey's post‐hoc test, n = 5, *p < 0.05 compared with the UAC+AAV2‐siControl group. NS, normal saline; UAC, unilateral anterior crossbite.

UAC model was constructed in rats after local injection of AAV2 in TMJ capsule for 2 weeks. The condyles were harvested 3 or 6 weeks after the UAC construction (**Figure** **7**A). The inhibitory effect of AAV2‐siVIM on VIM in the condylar cartilage of the UAC rats was confirmed by immunofluorescence (Figure 7B,C). Then, the histological analyses were further performed to investigate the effect of VIM inhibition on TMJOA in rats (Figure 7D). VIM inhibition recovered the decrease in cartilage thickness in the UAC+AAV2‐siControl group (Figure 7E), and the OARSI scores decreased after the inhibition of VIM (Figure 7F). In micro‐CT detection, the AAV2‐siControl group showed the densest trabeculae, whereas the UAC+AAV2‐siControl group showed clear hollow trabeculae. Trabecular density recovered significantly after the inhibition of VIM (Figure 7G). Further parametric analyses showed that the UAC+AAV2‐siControl group had lower BV/TV, Tb.N, and BMD, and higher BS/BV and Tb.Sp than the AAV2‐siControl group. However, the inhibition of VIM recovered the changes in the UAC group by inducing an increase in BV/TV, Tb.N, and BMD and a decrease in BS/BV and Tb.Sp (Figure 7H–L).

### 3.8 Inhibition of VIM Downregulated MMPs Expression and Inhibited MAPK Pathway In vivo

**Figure 8:**
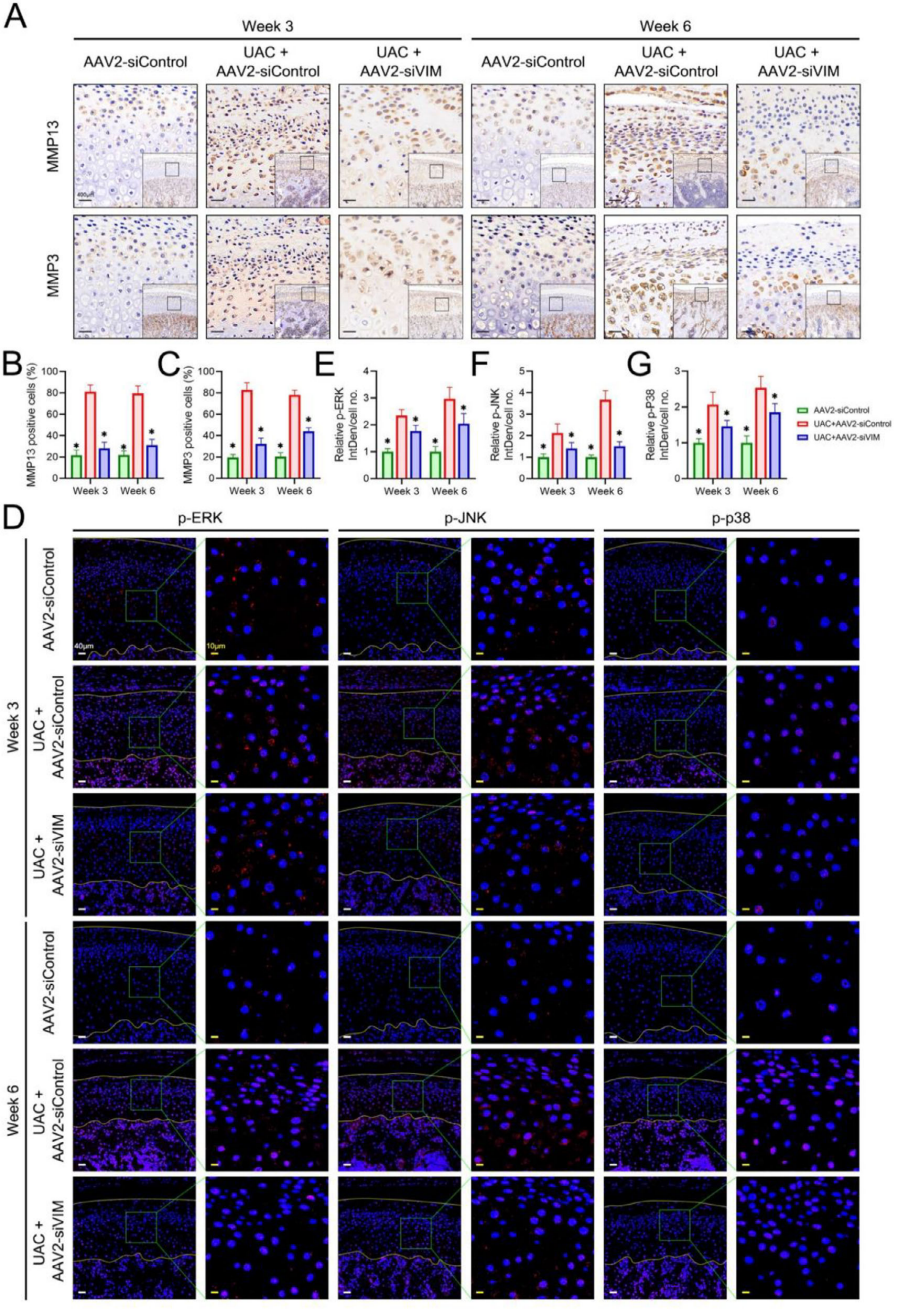
Effect of VIM inhibition in rats on MMP13, MMP3, and MAPK pathway. A) Histopathological analysis showing the effect of VIM inhibition on MMP13 and MMP3 in vivo. The percentage of MMP13‐positive cells (B) and MMP3‐positive cells (C) in the condylar cartilage of rats among various treatment groups was analyzed. Data is expressed as mean ± SD and analyzed using one‐way ANOVA followed by Tukey's post‐hoc test, n = 5, *p < 0.05 compared with the UAC+AAV2‐siControl group. D) The phosphorylation levels of ERK, JNK, and p38 in condylar cartilage of rats were detected by immunofluorescence, with the yellow lines showing the articular surface and the interface between bone and cartilage. Quantification of the phosphorylation levels of ERK (E), JNK (F), and p38 (G) in condylar cartilage of rats. Data is expressed as mean ± SD and analyzed using one‐way ANOVA followed by Tukey's post‐hoc test, n = 5, *p < 0.05 compared with the UAC+AAV2‐siControl group.

To further confirm the mechanism of VIM inhibition, IHC staining was performed. It showed that MMP13 and MMP3 were expressed in all the layers of condylar cartilage in the UAC+AAV2‐siControl groups, and were only localized in the mature layer and some of the hypertrophic layer in the condylar cartilage of rats treated with AA2‐siVIM (**Figure** **8**A). It showed that the proportion of cells expressing MMP13 and MMP3 was higher in the UAC+AAV2‐siControl groups than in the AAV2‐siControl group, but fewer condylar cells expressed these two markers in the AAV2‐siVIM treatment group (Figure 8B,C). Moreover, the phosphorylation of ERK, JNK, and p38 was activated after UAC construction, and was recovered in the AAV2‐siVIM treatment group (Figure 8D–G).

## 4 Discussion

TMJOA is a degenerative disease with a high incidence, and has recently received increasing attention.^[^ ^1^, ^2^ ^]^ As one of the most important components of TMJ, SF has been extensively studied by many researchers, and proteomic analysis has indicated the presence of 135–575 proteins in SF in patients with TMJOA.^[^ ^32^ ^]^ However, compared with previous studies, our study had a considerably larger sample size and identified a significantly higher number of proteins, which provides an insight into cartilage degeneration and subchondral bone disruption in TMJOA at the protein level. Our findings demonstrated a downregulation of ORM1 in patients with TMJOA and in the condylar cartilage of UAC rats. Moreover, the ORM1 level was lower in patients complaining of pain than in those without pain, which is a notable finding that deserves further exploration.

ORM1 is a key plasma protein with diverse physiological roles, including immune modulation, binding, and transport; maintenance of the capillary barrier; and metabolic regulation.^[^ ^33^ ^]^ In human plasma, the normal level of ORM is 0.5 mg mL^−1^ and can increase to 1–2.5 mg mL^−1^ in the presence of inflammation or tumors.^[^ ^33^, ^34^ ^]^ Nonetheless, it is worth noting that patients with active knee OA have lower serum ORM1 levels than those with nonactive OA.^[^ ^15^ ^]^ Although mainly produced by the liver and found in the serum, previous studies have investigated the ORM1 levels in the nervous system,^[^ ^35^ ^]^ alveolar macrophages,^[^ ^36^ ^]^ and oviduct.^[^ ^37^ ^]^ However, to our knowledge, the role of ORM1 in the condylar cartilage during TMJOA has not yet been reported. In our study, the presence of ORM1 in the condylar cartilage was confirmed, and the mechanism was further explored, but the uncertainty about the source of ORM1 needs to be further explored, which is a limitation of the present study.

Several studies have confirmed the anti‐inflammatory function of ORM1, for example, protecting against lethal shock induced by TNFα,^[^ ^38^ ^]^ inhibiting bacterial growth,^[^ ^39^ ^]^ binding to bacterial lipopolysaccharide to prevent its toxicity,^[^ ^40^ ^]^ and downregulating both IFN‐γ and TNF‐α.^[^ ^41^ ^]^ In our study, ORM1 showed an inhibitory effect on chemokines, including CXCL5 and CXCL8. Thus, ORM1 may relieve the inflammation by reducing the recruitment of pro‐inflammatory cells. However, this finding needs to be confirmed by further research. Apart from the previous research that indicated an association between ORM1 and inflammation, there is a lack of studies about the effect of ORM1 on cartilage matrix homeostasis. Bulk RNA sequencing was used to explore the role of ORM1 in chondrocytes, and it showed that ORM1 treatment downregulated MMPs. Consistent with our findings, Wang et al.^[^ ^42^ ^]^ reported an upregulation of MMPs and tissue inhibitors of metalloproteinases (TIMPs) in adipose tissues in ORM1‐deficient mice. MMPs are the key contributors to ECM generation in TMJOA, especially MMP13 and MMP3.^[^ ^43^ ^]^ Thus, these two MMPs were mainly studied. Further assays showed that ORM1 decreased the expression of MMP13 and MMP3 in inflammation‐induced chondrocytes. To further confirm the effect of ORM1 on TMJOA, the UAC model was used to induce TMJOA in rats. Consistent with the previous studies,^[^ ^23^ ^]^ it showed the condylar cartilage degeneration in the UAC rats. After ORM1 treatment, the destruction of condylar cartilage was reduced, and the expression of MMP13 and MMP3 decreased. These findings confirmed the inhibitory effect of ORM1 on MMP13 and MMP3.

To investigate the mechanism of ORM1 in regulating MMP expression, coIP and LC‐MS/MS were used to identify the downstream target of ORM1. Notably, VIM was identified as the only co‐immunoprecipitated protein of ORM1 at ≈50 kDa. VIM is a type III intermediate filament cytoskeletal protein, closely associated with inflammation and fibrosis.^[^ ^31^, ^44^ ^]^ Cartilage fibrosis is an important characteristic of OA, which indicates cartilage degradation and disruption of cartilage homeostasis.^[^ ^45^ ^]^ Notably, ORM1 has been reported to have an anti‐fibrotic effect by downregulating fibrosis‐related genes.^[^ ^42^ ^]^ Moreover, the imbalance between MMPs and TIMPs contributes to fibrosis, and MMP3 exhibits a distinct profibrotic role.^[^ ^46^ ^]^ These findings indicated that ORM1 has anti‐fibrotic effects on the cartilage by regulating its downstream targets: VIM and MMPs. In the inflammation response, VIM also plays an important role in immune cell functions such as migration, extravasation, homing, and target recognition.^[^ ^44^, ^47^ ^]^ VIM/ERK signaling is essential in inflammation. Xue et al.^[^ ^48^ ^]^ found that stimulated astrocytes showed the release of pro‐inflammatory cytokines via VIM/ERK signaling. Gong et al.^[^ ^49^ ^]^ reported that NLRP3 inflammasome can be activated via VIM/ERK/NF‐κB signaling. Further studies showed the direct interaction of VIM and ERK,^[^ ^31a^ ^]^ which plays an essential role in the regulation of ERK phosphorylation.^[^ ^50^ ^]^ Moreover, it has been reported that VIM contributes to the phosphorylation of JNK,^[^ ^31c^ ^]^ and p38.^[^ ^50b^ ^]^ Previous studies have shown that MAPKs promote the expression of MMPs in TMJOA.^[^ ^51^ ^]^ Thus, ORM1 may alleviate TMJOA by suppressing the VIM/MAPK/MMP signaling.

To confirm this hypothesis, further assays showed that ORM1 inhibited VIM, and inhibited the phosphorylation of ERK, JNK, and p38. Then, consistent with previous findings,^[^ ^31^, ^48^, ^49^, ^50^ ^]^ the important role of VIM in the phosphorylation of ERK, JNK, and p38 was confirmed. The expression of MMP13 and MMP3 was downregulated after the inhibition of ERK, JNK, and p38, which has been widely demonstrated in several studies.^[^ ^51^ ^]^ Moreover, the effect of breaking the ORM1‐VIM binding on the activities of MAPK pathway and the MMPs expression was further explored. It showed that the inhibition effect of ORM1 on the MAPK pathway and the MMPs expression depends on the ORM1‐VIM binding. The role of ORM1 may be similar to that of the commonly used VIM inhibitor withaferin A, whose mechanism of action includes direct binding to VIM.^[^ ^52^ ^]^ However, the in‐depth mechanism underlying the downregulation of VIM needs further exploration. Finally, to confirm the role of VIM in TMJOA, AAV2 was used to inhibit VIM in UAC rats, and the inhibition of VIM also showed a protective effect on condylar cartilage homeostasis during TMJOA.

## 5 Conclusion

**Figure 9:**
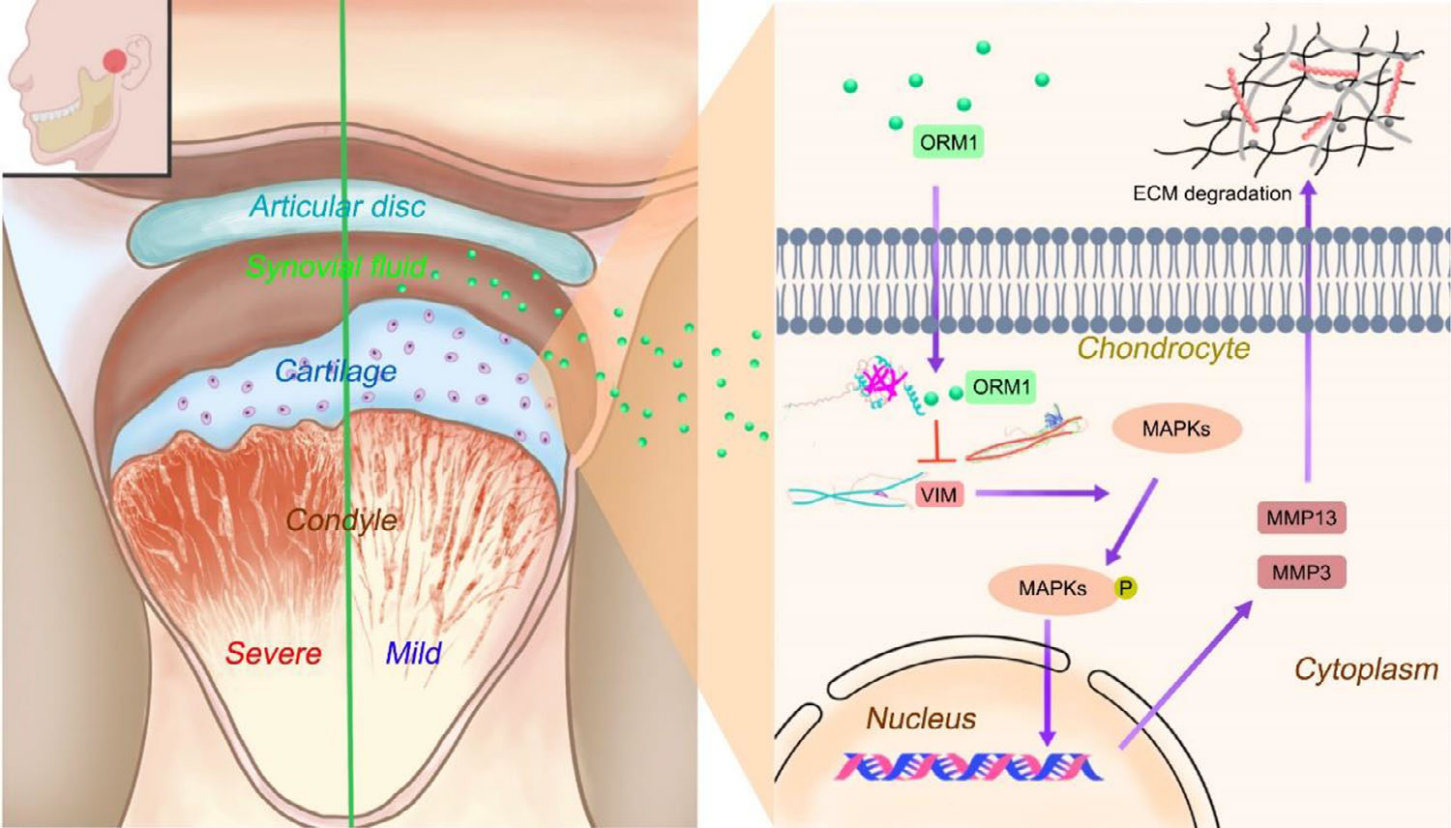
Schematic diagram of the role of ORM1 in maintaining cartilage homeostasis via suppressing VIM/MAPK/MMP signaling.

In conclusion, the protective effect of ORM1 on the condylar cartilage during TMJOA was confirmed and systematically analyzed for the first time. Our study demonstrated that ORM1 is downregulated in patients and rats with TMJOA. Further assays showed that ORM1 inhibits the phosphorylation of MAPK (ERK, JNK, and p38) pathway via directly binding to and decreasing VIM, thereby downregulating the expression of MMP13 and MMP3, and preventing cartilage matrix degradation during TMJOA. The present study demonstrates the important role of ORM1 in maintaining cartilage homeostasis via the suppression of VIM/MAPK/MMP signaling and suggests that ORM1 is a promising target for therapeutic intervention in TMJOA (**Figure** **9**).

## Conflict of Interest

The authors declare no conflict of interest.

## Author Contributions

D.Z. and Y.Z. are co‐first authors. C.Y. contributed to conception and critically revised the manuscript; P. S. contributed to design and critically revised the manuscript; D. Z. contributed to acquisition, analysis, and interpretation, and drafted the manuscript; Y.Z. contributed to acquisition and analysis, and drafted the manuscript; S.X. contributed to acquisition and analysis; C.L. contributed to acquisition and drafted the manuscript. All authors gave final approval and agreed to be accountable for all aspects of work, ensuring integrity and accuracy.

## Supporting information



Supporting Information

## Data Availability

The data that support the findings of this study are available from the corresponding author upon reasonable request.
