# Supplementary material for: Orosomucoid 1 Ameliorates Temporomandibular Joint Osteoarthritis by Maintaining Cartilage Homeostasis
Source: Adv Sci (Weinh). 2025 Jun 29;12(36):e00028. doi: 10.1002/advs.202500028 (PMC12463022; doi:10.1002/advs.202500028)
Supplement: Supplementary file 1 — Supporting Information [file ADVS-12-e00028-s001.docx]

**Supplementary Materials**

**This file includes**

Figures S1 to S8;

Tables S1 to S4.


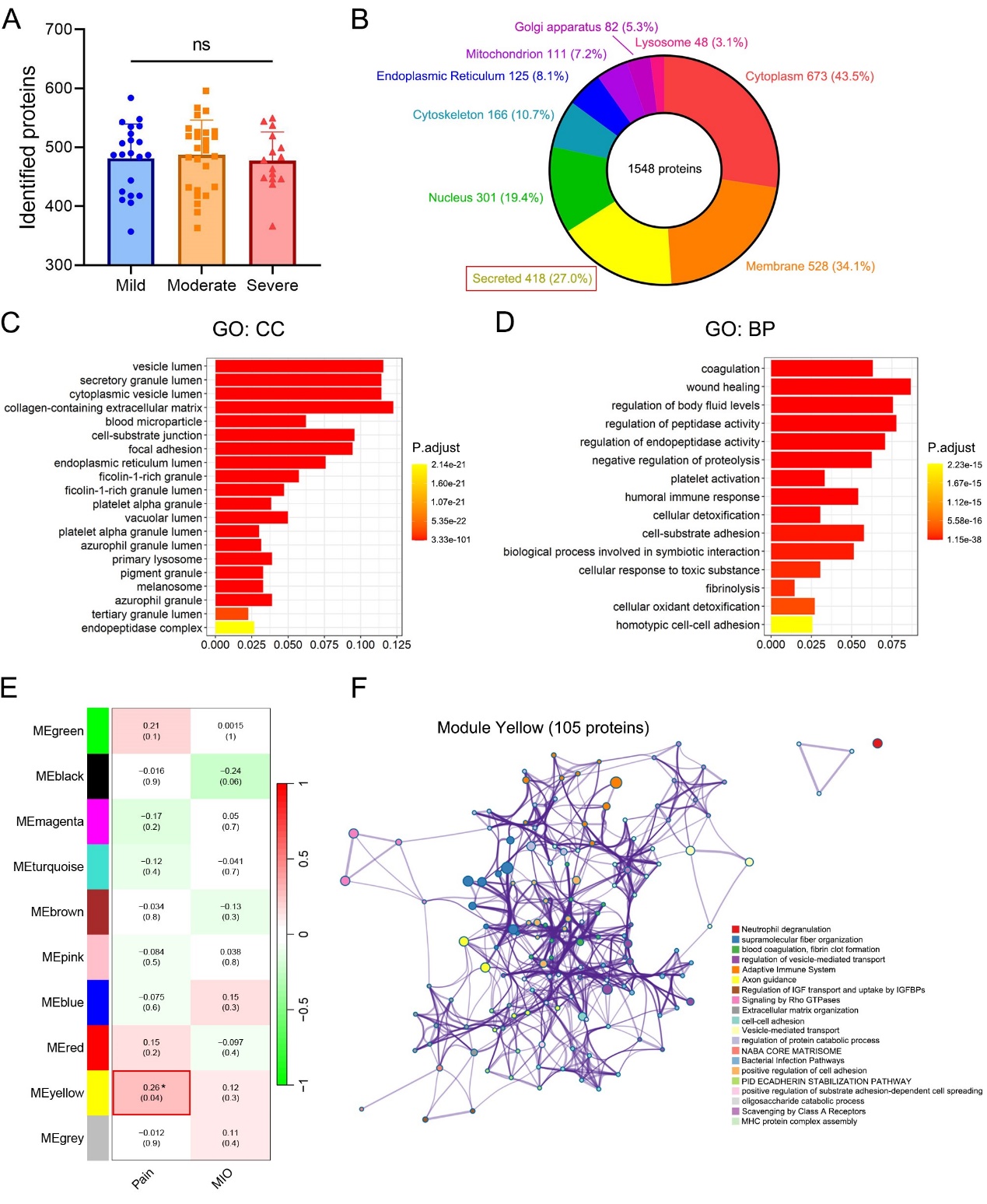


**Supplementary Figure 1.** Further proteomic analysis of synovial fluid (SF) samples. (A) Number of identified proteins in the samples with different TMJOA grades, including Mild (n=21), Moderate (n=27), and Severe (n=15) groups. Data is expressed as mean ± SD and analyzed using one-way analysis of variance (ANOVA) followed by Tukey’s post-hoc test; ns, not significant. (B) Subcellular locations of the identified proteins. Gene ontology (GO) analysis identifying the Cellular component (C) and Biological Process (D) terms associated with the proteins. (E) Weighted gene co-expression network analysis (WGCNA) results showing the association between the protein modules and pain or maximum interincisal opening (MIO). (F) Pathway analysis by Metascape in genes in module Yellow.


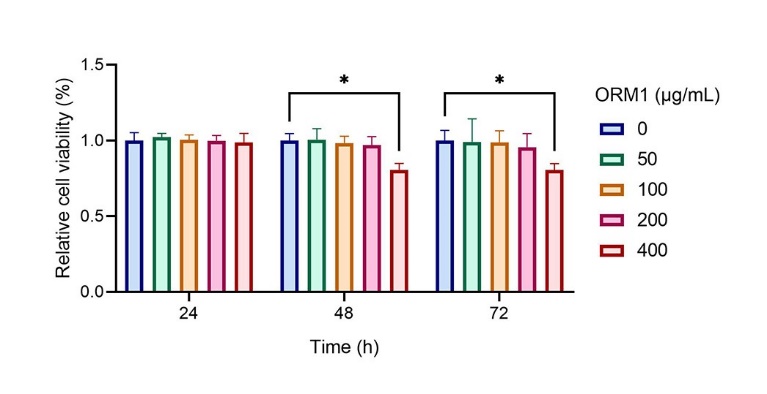


**Supplementary Figure 2.** Cell proliferation assay of C28/I2 cells treated with different concentration of ORM1 protein. Data is expressed as mean ± SD and analyzed using one-way ANOVA followed by Tukey’s post-hoc test, n=3, * p < 0.05 compared with the 0 μg/mL ORM1 group.


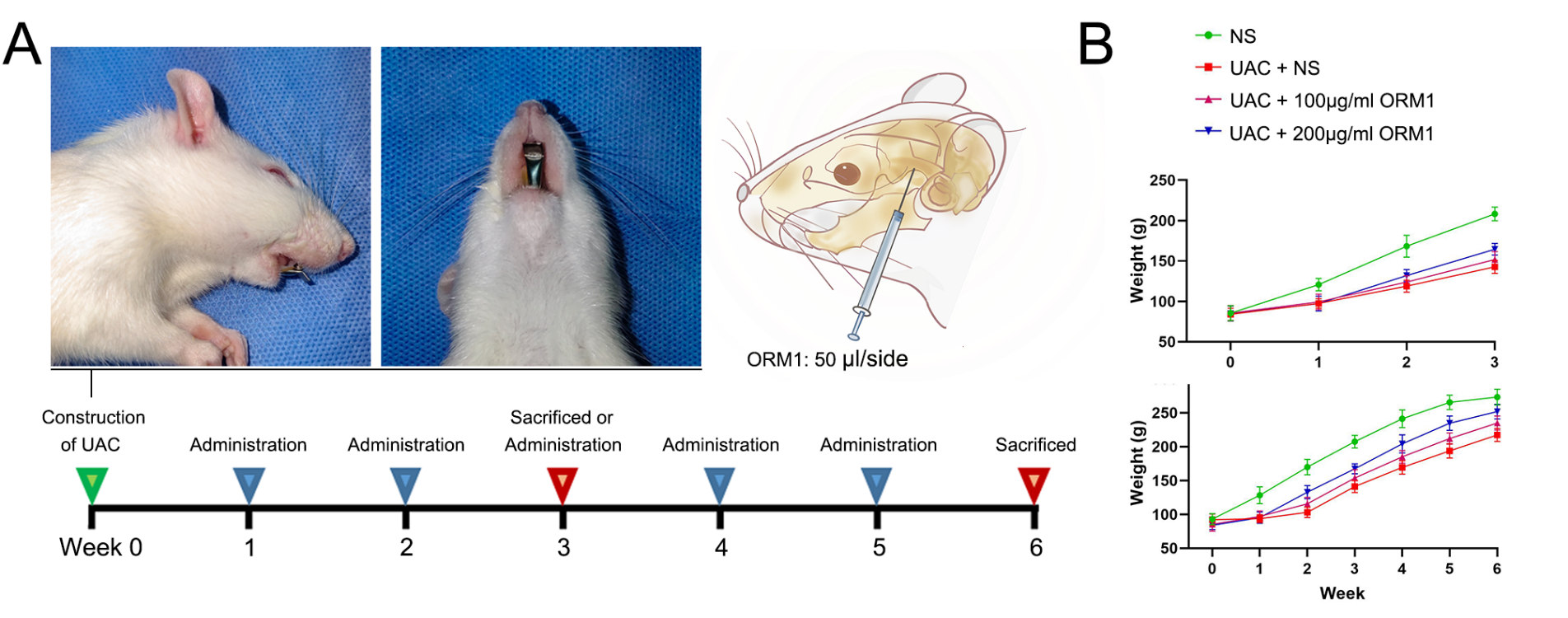


**Supplementary Figure 3.** Construction of the unilateral anterior crossbite (UAC) model in rats. (A) Images of UAC construction and schematic diagram depicting the administration; (B) Weight of the rats in different groups. Data is expressed as mean ± SD; Weight of the rats at week 3 and week 6 were analyzed using one-way ANOVA followed by Tukey’s post-hoc test, n=5.


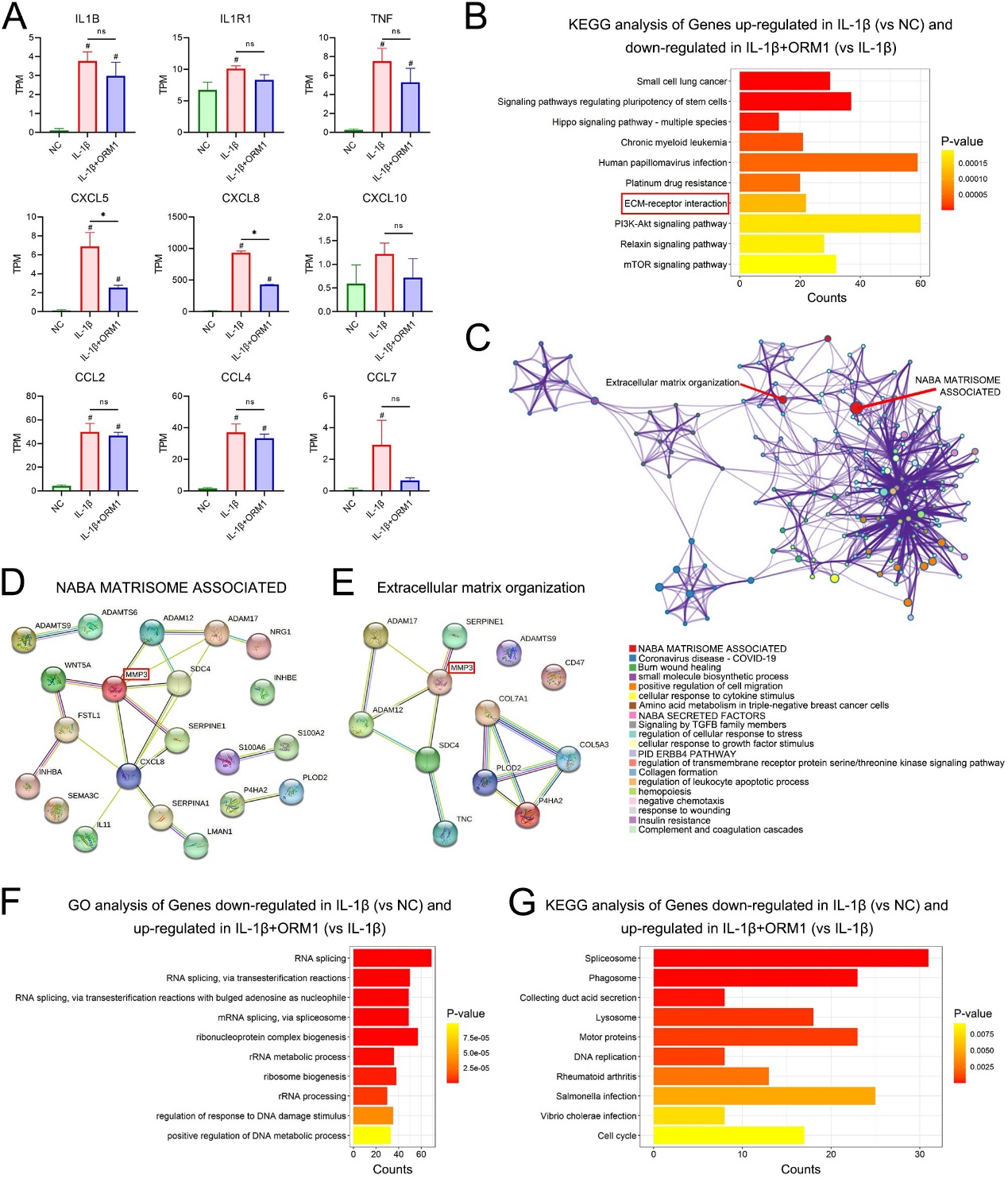


**Supplementary Figure 4.** Further bulk RNA sequencing of C28/I2 cells. (A) Expression levels (in TPM) of inflammation-related genes in the three treatment groups. Data is expressed as mean ± SD and analyzed using one-way ANOVA followed by Tukey’s post-hoc test, n=3; ns not significant, * p < 0.05, ^#^ p < 0.05 compared with the NC group. (B) KEGG pathway analysis of genes that were upregulated after IL-1β treatment and downregulated after ORM1 treatment. (C) Pathway analysis by Metascape of genes that were upregulated after IL-1β treatment and downregulated after ORM1 treatment. (D) PPI network of analyzed genes in NABA matrisome-associated pathway. (E) PPI network of analyzed genes in extracellular matrix organization pathway. Gene ontology (GO) (F) and Kyoto Encyclopedia of Genes and Genomes (KEGG) pathway (G) analysis of genes that were downregulated after IL-1β treatment and upregulated after ORM1 treatment.


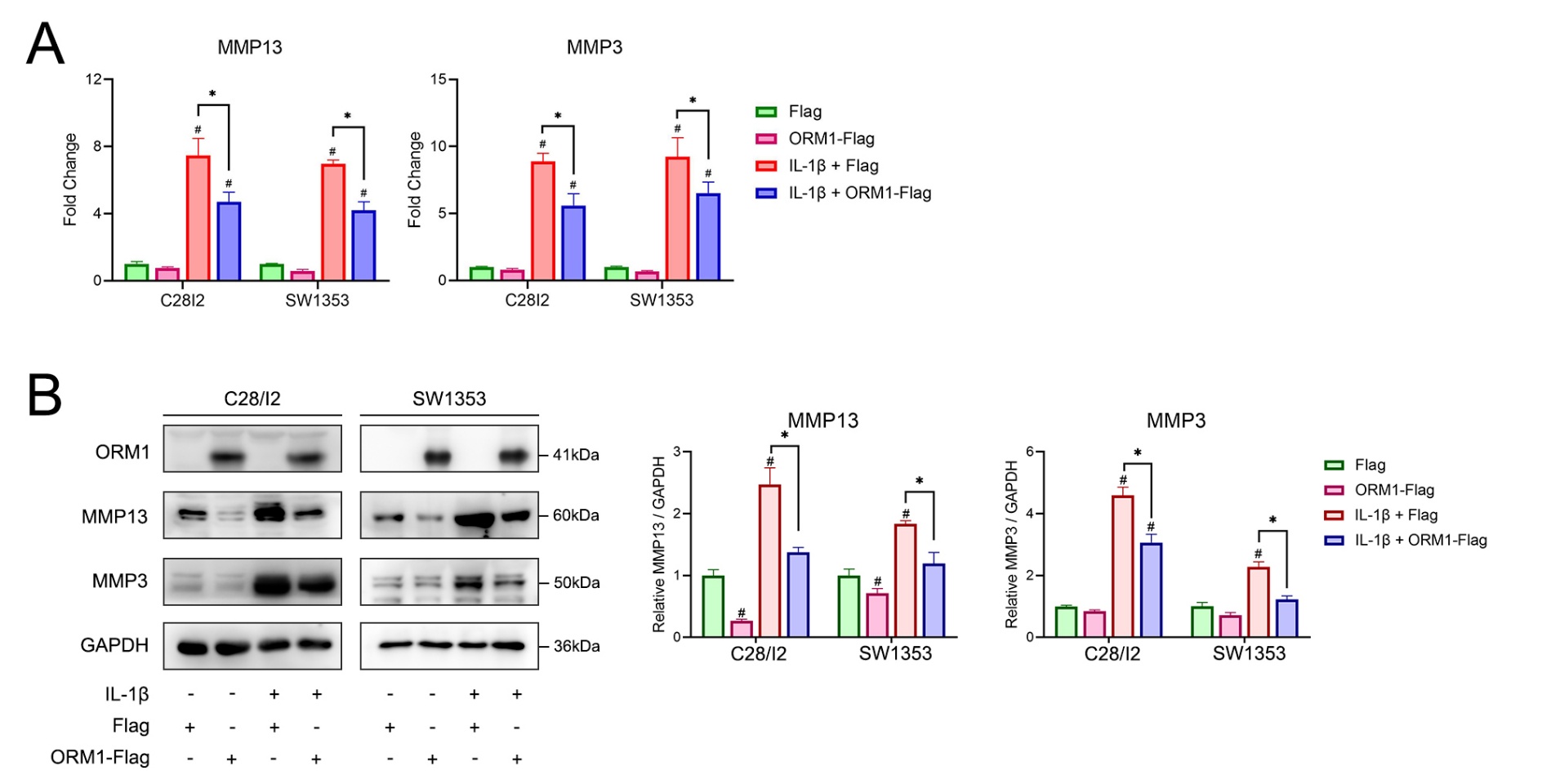


**Supplementary Figure 5.** Changes in the expression of MMP13 and MMP3 in cells after overexpression of ORM1. (A) mRNA expression level of MMP13 and MMP3 in both natural and inflammation environments in C28/I2 and SW1353 cells after transfected with ORM1-Flag plasmid. Data is expressed as mean ± SD and analyzed using one-way ANOVA followed by Tukey’s post-hoc test, n=3, * p < 0.05, ^#^ p < 0.05 compared with the Flag group. (B) Protein level of MMP13 and MMP3 in both natural and inflammation environments in C28/I2 and SW1353 cells after transfected with ORM1-Flag plasmid. Data is expressed as mean ± SD and analyzed using one-way ANOVA followed by Tukey’s post-hoc test, n=3, * p < 0.05, ^#^ p < 0.05 compared with the Flag group.


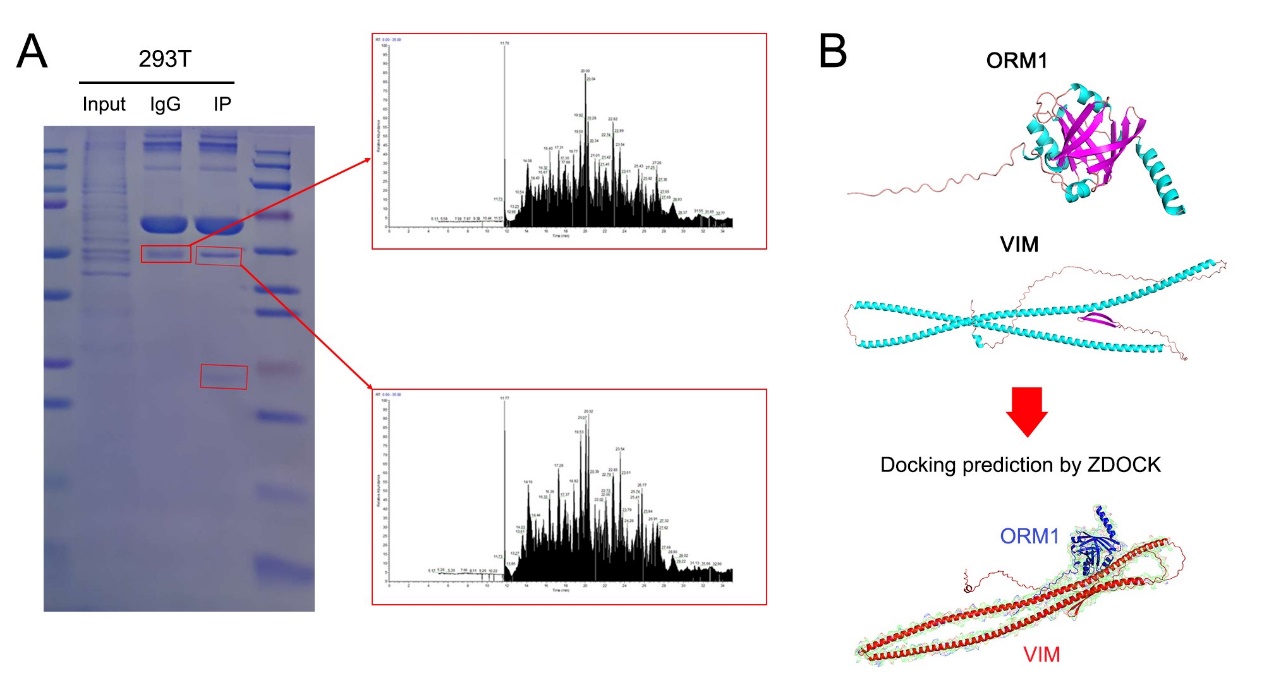


**Supplementary Figure 6.** Identification of the interaction between ORM1 and VIM. (A) Co-immunoprecipitation (Co-IP) and liquid chromatography–tandem mass spectrometry (LC-MS/MS) identifying the co-immunoprecipitated ORM1 protein at around 50 kDa. (B) Docking between ORM1 and VIM predicted by ZDOCK.


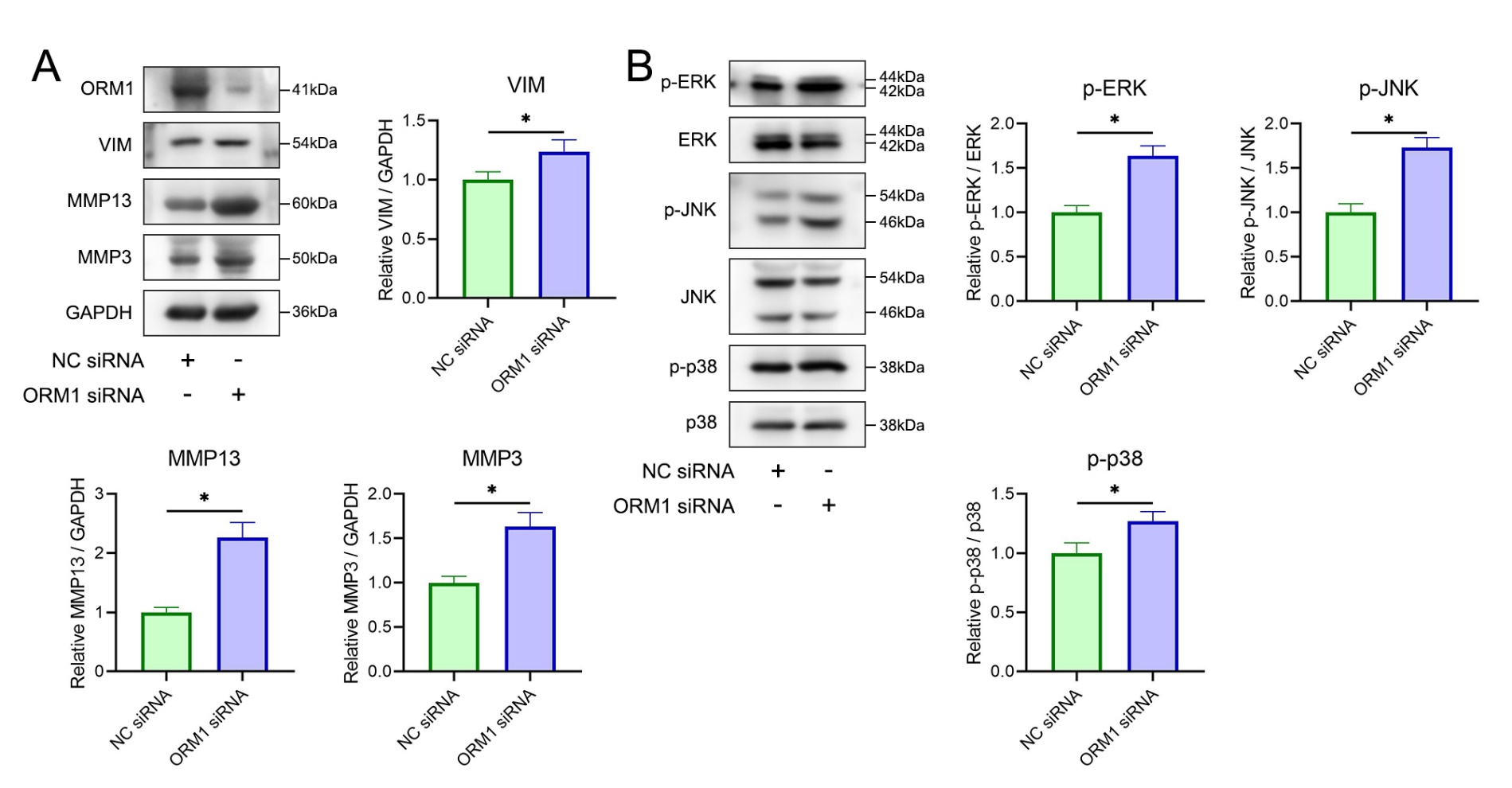


**Supplementary Figure 7.** The effect of ORM1 inhibition in C28/I2 cells. (A) Expression of MMP13 and MMP3 after knockdown of ORM1 in C28/I2 cells. Data are expressed as mean ± SD and analyzed using unpaired Student's t test (two-tailed), n=3, * *p* < 0.05. (B) The phosphorylation of ERK, JNK, and p38 after knockdown of ORM1 in C28/I2 cells. Data are expressed as mean ± SD and analyzed using unpaired Student's t test (two-tailed), n=3, * *p* < 0.05.


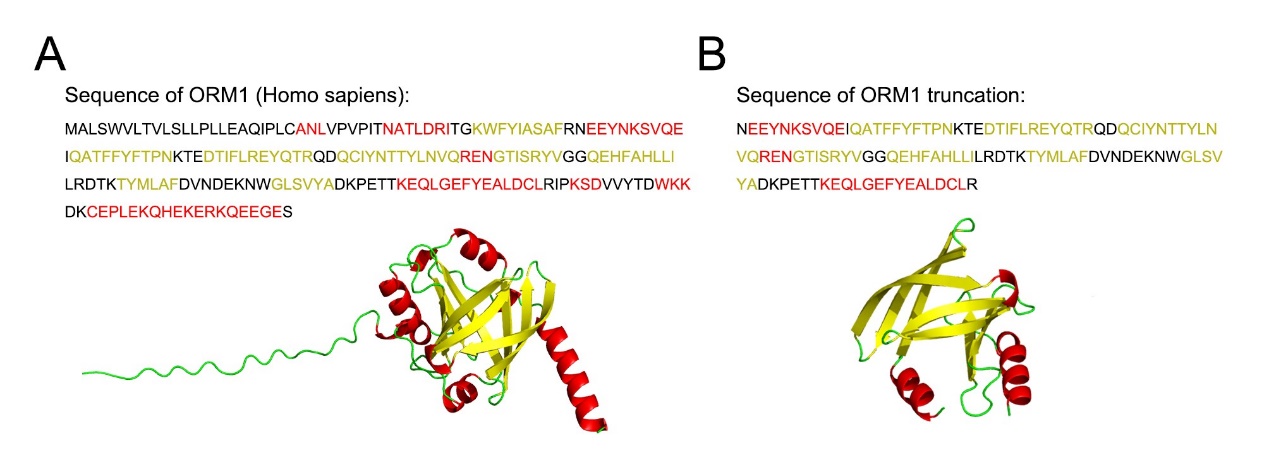


**Supplementary Figure 8.** Sequence of ORM1 and ORM1 truncation. (A) Sequence of ORM1. (B) Sequence of the 52 to 167 amino acid truncation of ORM1. The red parts represent the alpha-helix structure, and the yellow part represent the beta-sheet structure.

**Supplementary Table 1.** Patient cohort

| **No.** | **Gender** | **Age** | **CR grade** | **Bruxism** | **Pain (VAS)** | **MIO, mm** |
| --- | --- | --- | --- | --- | --- | --- |
| 14 | F | 20 | 3 | Y | 3 | 41 |
| 15 | F | 20 | 2 | N | 1 | 37 |
| 16 | M | 20 | 3 | Y | 0 | 45 |
| 17 | M | 20 | 1 | N | 0 | 32 |
| 18 | F | 19 | 3 | Y | 2 | 25 |
| 19 | M | 19 | 1 | Y | 1 | 40 |
| 20 | F | 19 | 2 | Y | 5 | 30 |
| 21 | M | 19 | 2 | N | 2 | 48 |
| 22 | F | 18 | 2 | N | 0 | 18 |
| 23 | F | 18 | 2 | N | 0 | 30 |
| 24 | F | 17 | 2 | Y | 1 | 45 |
| 25 | F | 17 | 2 | N | 2 | 35 |
| 26 | F | 17 | 2 | N | 0 | 35 |
| 27 | F | 17 | 3 | Y | 3 | 30 |
| 28 | M | 17 | 1 | N | 2 | 36 |
| 29 | F | 17 | 3 | Y | 2 | 28 |
| 30 | F | 16 | 2 | Y | 1 | 42 |
| 31 | F | 16 | 3 | Y | 2 | 30 |
| 32 | F | 16 | 2 | N | 2 | 28 |
| 33 | F | 16 | 2 | N | 0 | 37 |
| 34 | F | 16 | 2 | Y | 3 | 30 |
| 35 | F | 15 | 1 | N | 3 | 15 |
| 36 | F | 14 | 3 | N | 3 | 40 |
| 37 | F | 13 | 2 | Y | 2 | 45 |
| 38 | F | 13 | 2 | N | 3 | 25 |
| 39 | F | 13 | 3 | N | 0 | 25 |
| 40 | F | 13 | 3 | Y | 2 | 20 |
| 41 | F | 13 | 1 | Y | 0 | 29 |
| 47 | F | 16 | 3 | - | - | 20 |
| 49 | F | 15 | 2 | - | 0 | 40 |
| 50 | F | 13 | 3 | - | - | 38 |
| 51 | F | 19 | 2 | Y | 1 | - |
| 52 | F | 17 | 3 | Y | 1 | 37 |
| 53 | F | 17 | 1 | - | - | 21 |
| 54 | F | 14 | 1 | - | 0 | 38 |
| 55 | F | 15 | 1 | N | 0 | 30 |
| 56 | F | 17 | 2 | - | 0 | 37 |
| 58 | F | 18 | 1 | - | 0 | 45 |
| 59 | M | 13 | 2 | - | - | 37 |
| 61 | F | 17 | 1 | N | 5 | 37 |
| 64 | M | 20 | 1 | Y | 5 | 43 |
| 65 | F | 17 | 2 | N | 3 | - |
| 66 | F | 16 | 2 | - | 0 | 40 |
| 67 | M | 16 | 3 | - | - | 45 |
| 68 | F | 16 | 1 | Y | 3 | - |
| 69 | F | 19 | 2 | N | 0 | 37 |
| 73 | F | 14 | 2 | Y | 7 | 35 |
| 75 | M | 18 | 3 | - | - | - |
| 78 | F | 15 | 2 | Y | 3 | 21 |
| 79 | F | 20 | 2 | Y | 0 | 35 |
| 84 | F | 15 | 2 | Y | 1 | 40 |
| 86 | F | 14 | 3 | Y | 0 | 48 |
| 88 | F | 15 | 1 | N | 0 | 28 |
| 89 | F | 15 | 1 | Y | 0 | 40 |
| 93 | F | 15 | 1 | N | 0 | 37 |
| 95 | F | 12 | 2 | Y | 0 | 34 |
| 97 | M | 17 | 1 | - | 0 | 50 |
| 98 | F | 19 | 1 | - | 0 | 41 |
| 99 | F | 20 | 1 | - | - | 35 |
| 101 | F | 13 | 1 | N | 0 | 35 |
| 103 | F | 19 | 1 | N | 0 | 20 |
| 104 | F | 19 | 2 | N | 1 | 32 |
| 106 | F | 18 | 1 | Y | 0 | 32 |

CR grade: Condylar resorption grade, 1=Mild, 2=Moderate, 3=Severe

VAS: Visual analogue scale

MIO: Maximum inter-incisal opening

F: Female

M: Male

Y: Yes

N: No

-: lost to follow-up

**Supplementary Table 2.** Primers used in reverse transcription quantitative PCR

| **Names** | **Species** | **Sequence (5′ to 3′)** |
| --- | --- | --- |
| MMP13 | Homo sapiens | Forward: ACTGAGAGGCTCCGAGAAATG  Reverse: GAACCCCGCATCTTGGCTT |
| MMP3 | Homo sapiens | Forward: GAGCTGGATACCCAAGAGGC  Reverse: AGCCTGGCTCCATGGAATTT |
| GAPDH | Homo sapiens | Forward: AGAAGGCTGGGGCTCATTT  Reverse: GGTGCTAAGCAGTTGGTGGT |

**Supplementary Table 3.** The siRNA sequences

| **Names** | **Species** | sense（5'-3'） | antisense（5'-3'） |
| --- | --- | --- | --- |
| ORM1 siRNA | *Homo sapiens* | CCAAGUCAGAUGUCGUGUATT | UACACGACAUCUGACUUGGTT |
| VIM siRNA | *Homo sapiens* | GCAGAAGAAUGGUACAAAUTT | AUUUGUACCAUUCUUCUGCTT |
| NC siRNA | *Homo sapiens* | UUCUCCGAACGUGUCACGUTT | ACGUGACACGUUCGGAGAATT |

**Supplementary Table 4.** The antibodies used in western blot

| **Antibody** | **Catalog No.** | **Manufacturer** | **Dilution ratio** |
| --- | --- | --- | --- |
| Anti-GAPDH antibody [6C5] - Loading Control | ab8245 | Abcam | 1:5000 |
| Anti-MMP13 antibody | ab39012 | Abcam | 1:1500 |
| Anti-MMP3 antibody [EP1186Y] | ab52915 | Abcam | 1:1500 |
| Anti-Vimentin antibody [EPR3776] - Cytoskeleton Marker | ab92547 | Abcam | 1:1500 |
| ORM1 Monoclonal Antibody (A2-B10) | MA5-32836 | Invitrogen | 1:1500 |
| p44/42 MAPK (Erk1/2) (137F5) Rabbit mAb | 4695 | Cell Signaling Technology | 1:1500 |
| SAPK/JNK Antibody | 9252 | Cell Signaling Technology | 1:1500 |
| p38 MAPK (D13E1) XP® Rabbit mAb | 8690 | Cell Signaling Technology | 1:1500 |
| Phospho-p44/42 MAPK (Erk1/2) (Thr202/Tyr204) (D13.14.4E) XP® Rabbit mAb | 4370 | Cell Signaling Technology | 1:1500 |
| Phospho-SAPK/JNK (Thr183/Tyr185) (81E11) Rabbit mAb | 4668 | Cell Signaling Technology | 1:1500 |
| Phospho-p38 MAPK (Thr180/Tyr182) (D3F9) XP® Rabbit mAb | 4511 | Cell Signaling Technology | 1:1500 |
| Anti-rabbit IgG, HRP-linked Antibody | 7074 | Cell Signaling Technology | 1:5000 |
| Anti-mouse IgG, HRP-linked Antibody | 7076 | Cell Signaling Technology | 1:5000 |
